# Universal chiral-triggered magnetization switching in confined nanodots

**DOI:** 10.1038/srep10156

**Published:** 2015-06-10

**Authors:** Eduardo Martinez, Luis Torres, Noel Perez, Maria Auxiliadora Hernandez, Victor Raposo, Simone Moretti

**Affiliations:** 1 Universidad de Salamanca. Plaza de los Caidos s/n, E-37008, Salamanca. Spain

## Abstract

Spin orbit interactions are rapidly emerging as the key for enabling efficient current-controlled spintronic devices. Much work has focused on the role of spin-orbit coupling at heavy metal/ferromagnet interfaces in generating current-induced spin-orbit torques. However, the strong influence of the spin-orbit-derived Dzyaloshinskii-Moriya interaction (DMI) on spin textures in these materials is now becoming apparent. Recent reports suggest DMI-stabilized homochiral domain walls (DWs) can be driven with high efficiency by spin torque from the spin Hall effect. However, the influence of the DMI on the current-induced magnetization switching has not been explored nor is yet well-understood, due in part to the difficulty of disentangling spin torques and spin textures in nano-sized confined samples. Here we study the magnetization reversal of perpendicular magnetized ultrathin dots, and show that the switching mechanism is strongly influenced by the DMI, which promotes a universal chiral non-uniform reversal, even for small samples at the nanoscale. We show that ultrafast current-induced and field-induced magnetization switching consists on local magnetization reversal with domain wall nucleation followed by its propagation along the sample. These findings, not seen in conventional materials, provide essential insights for understanding and exploiting chiral magnetism for emerging spintronics applications.

Understanding and controlling the current-induced magnetization dynamics in high perpendicular magnetocristaline anisotropy heterostructures consisting of a heavy-metal (HM), a ferromagnet (FM) and an oxide (HM/FM/O) or asymmetric HM1/FM/HM2 stacks, is nowadays the focus of active research^1^,^2^,^3^,^4^,^5^,^6^,^7^,^8^,^9^,^10^,^11^,^12^,^13^,^14^,^15^,^16^. Apart from their interest for promising spintronics applications, these systems are also attracting growing attention from a fundamental point of view due to the rich physics involved in the current-induced magnetization switching (CIMS)^1^,^2^,^3^,^4^,^5^ and in the current-induced domain wall motion (CIDWM)^7^,^8^,^9^,^10^,^11^. Indeed, the combination of a HM and a thin FM film gives rise to new phenomena which normally vanish in bulk, but play an important role as the thickness of the FM is reduced to atomistic size.

Current-induced torques arising from spin-orbit phenomena can efficiently manipulate magnetization. In particular, the Slonczewski-like spin-orbit torque (SL-SOT)^1^,^2^,^3^,^4^,^5^,^6^,^7^,^8^,^9^,^10^,^11^ can switch the magnetization from up (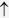) to down (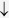) states and vice versa under the presence of small in-plane fields. The SL-SOT is expressed as


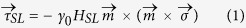


where 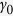 is the gyromagnetic ratio, 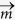 the unit vector along the magnetization, 
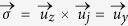
 the unit vector along the polarized current which is perpendicular to both the easy axis (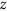) and current direction given by 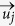, and 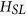 parameterizes the torque. CIMS in ultrathin Pt/Co/AlO, where the Co layer is only 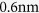 thick (around three atomic layers), was experimentally observed first by Miron and coworkers^1^, where the switching was attributed to SL-SOT due to the Rashba field^17^,^18^. The Rashba effect would generate both field-like (FL-SOT)^17^,^18^ and Slonczewski-like (SL-SOT)^19^,^20^ spin-orbit torques. Similar to the conventional spin transfer torque (STT)^21^, both Rashba FL and SL SOTs have magnitudes proportional to the spin polarization of the current (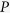) flowing through the FM, and therefore, they are expected to be negligible for an ultrathin FM, as reported in experimental studies^22^,^23^,^24^. Indeed, Liu *et al.*^3^ studied CIMS in Pt/Co/AlO, similar to the study by Miron *et al.*^1^ but they did not find any significant dominant Rashba FL torque, and therefore the Rashba contribution to the SL-SOT should be even vanishingly small. This was also the conclusion from switching experiments in asymmetric Pt/Co/Pt^8^ and for Pt/CoFe/MgO^9^. Instead of the Rashba SL-SOT, the switching is consistent with an alternative SL-SOT based on the spin Hall effect (SHE)^25^,^26^. The SL-SOT due to the SHE is physically distinct from other torques STTs and Rashba-SOTs: it is independent of 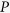 because it arises from the spin current generated in the HM, rather than the spin polarization of the charge current in the FM.

The key to the existence of the SOTs is a high spin-orbit coupling combined with structural inversion asymmetry (SIA) in these heterostructures: if the top and bottom interfaces/layers sandwiching the FM were completely symmetric, all the mentioned effects should cancel out. However, not only the SIA plays a role in these current-induced magnetization dynamics but, it can also influence the static magnetization state through the interfacial Dzyaloshinskii-Moriya interaction (DMI)^27^,^28^,^29^,^30^. In systems with SIA, the interfacial DMI is an anisotropic exchange contribution which directly competes with the exchange interaction, and when strong enough, it promotes non-uniform magnetization textures of a definite chirality such as spin helixes^31^, chiral domain walls (DWs)^8^,^9^,^10^,^11^,^30^ and skyrmions^32^,^33^,^34^. In particular, the experiments on current-induced DW motion along Pt/Co/AlO^7^ or Pt/CoFe/MgO^9^,^11^ can be explained by the combined action of the DMI and the SHE. The strong DMI in these Pt systems is the responsible of the formation of the Neel walls with a given chirality, which are driven by the SHE^9^,^10^,^11^. However, the influence of the DMI on the CIMS has not been explored nor is yet well-understood, due in part to the difficulty of disentangling spin torques and spin textures in nano-size confined dots.

On the other hand, experiments on CIMS in these asymmetric multilayers are usually interpreted in the framework of the single-domain model (SDM) which neglects both the exchange and DMI contributions, and only a few recent studies in extended samples at the microscale (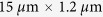) have considered the non-uniform magnetization by full 3D micromagnetic simulations^35^,^36^,^37^,^38^. Here we focus on CIMS of a ultrathin Pt/Co/AlO with in-plane dimensions two orders of magnitude below (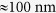). Although these dimensions should be amenable for the uniform magnetization description, our study indicates that the DMI is also essential to describe the CIMS at these dimensions, which occurs through chiral asymmetric DW nucleation and propagation. We analyze the key ingredients of the switching and confirm that a full micromagnetic analysis is necessary to describe and quantify the spin Hall angle under realistic conditions.

## Results

**Figure 1:**
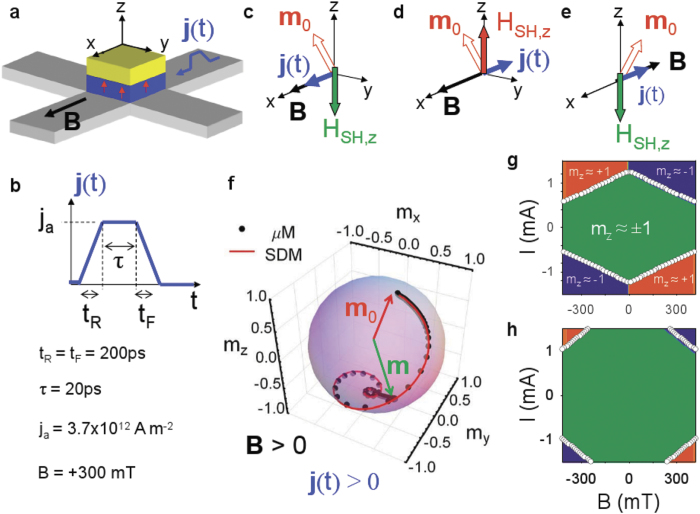
Current induced magnetization switching in the absence of DMI (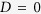) (a) Schematic representation of the analyzed heterostructure with the Co layer in blue. (b) Temporal variation of the density current pulses 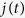 with rising (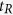), falling (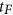) and duration (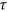) times. (c)-(e) Out-of-plane component of spin Hall effective field 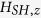 as a function of the applied field 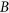 and density current 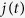 directions. (f) Magnetization trajectories starting from up state (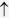) to down state (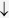) under a static field of 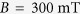 and a pulse with 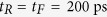, 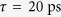 and 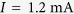 (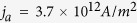 flowing through both the Pt and Co layers) for 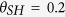. Solid red line depicts Single Domain Model (SDM) results whereas solid black dots correspond to full micromagnetic (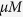) simulations in the absence of DMI (*D* = 0) for the averaged magnetization components over the sample volume (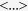). (g)-(h) Stability phase diagrams indicating the terminal out-of-plane magnetization component 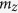 as a function of 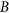 and 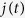 for 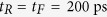, 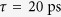, and 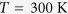 as computed with the SDM with a high 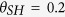 (g) and with realistic 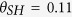 (h). Open circles denote the transition between switching and no-switching at zero temperature.

The considered heterostructure here consists on a thin ferromagnetic Co nanosquare with a side of 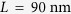 and a thickness of 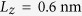 sandwiched between a AlO layer and on top of a Pt cross Hall (Fig. 1(a)). The thickness of the Pt layer is 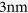. Typical high PMA material parameters were adopted in agreement with experimental values^5^,^6^,^38^. Details about the physical parameters can be found in Methods.

### Cuasi-uniform current-induced magnetization switching in the absence of the DMI: single domain approach and micromagnetic results

The current induced magnetization dynamics under static in-plane longitudinal field 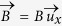 and current pulses 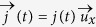 is studied from both Single Domain Model (SDM) and full micromagnetic Model (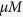) points of view (see Methods). We first review the CIMS in the framework of the SDM, where the magnetization is assumed to be spatially uniform (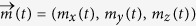). Within this approach the conventional symmetric exchange and interfacial DMI are not taken into account (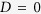). In the absence of in-plane fields (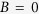) or thermal fluctuations, with the magnetization initially pointing along the easy 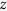-axis (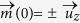, 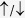), a moderate current 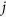 along the longitudinal direction (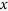-axis) only generates an effective SHE field along the 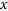-axis which does not promote the out - of-plane magnetization reversal (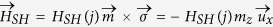). However, in the presence of a longitudinal field 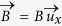 below the saturating in-plane field (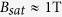), 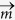 acquires a finite longitudinal component 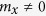 parallel to 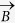, and the current pulse 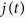 generates an out-of-plane component effective SHE field 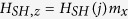. If 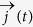 is parallel to 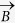 (either 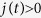 and 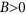 as in Fig. 1(c), or 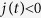 and 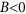 as in Fig. 1(e)), and their magnitudes are sufficiently strong, the magnetization is stabilized pointing parallel to the out-of-plane component of 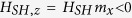, i.e. along the 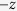-axis (Fig. 1(c) and (e)). On the contrary, if the field and the current pulse are anti parallel to each other (either 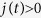 and 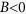, or 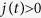 and 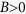), 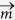 is stabilized along the 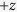-axis (Fig. 1(d)).

Fig. 1(f) shows the 3D magnetization trajectories for CIMS starting from the up state (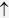) with 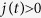 and 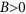 for 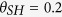 in the absence of DMI (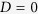). In this case, the reversal occurs via quasi-uniform magnetization precession, and therefore, the SDM reproduces accurately the magnetization dynamics (solid red line in Fig. 1(f)) computed from a full 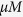 point of view (black dots in Fig. 1(f)), confirming the validity of the uniform magnetization approach in the absence of DMI (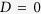).

The SDM stability phase diagrams showing the terminal out-of-plane magnetization direction as function of 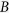 and 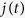 (with 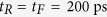, 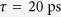 and different amplitudes 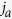) are depicted in Fig. 1(g) and (h) for a high 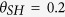 and a more realistic 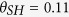 value of spin Hall angle respectively. These results were computed at room temperature by averaging over 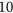 stochastic realizations. The same results were also obtained at zero temperature (see open circles in Fig. 1(g) and (h)). Note that 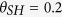 is around twice the value experimentally deduced for the Pt/Co from efficiency measurements^6^, where 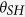 was estimated 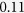. Therefore, these experiments^6^ cannot be reproduced by the SDM unless unrealistic values of 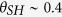 are assumed^6^. As it will be shown later, the key ingredient to achieve quantitative agreement is the presence of DMI, which can only be taken into account in a full 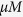 analysis.

### Non-uniform magnetization patterns and current induced magnetization switching (CIMS) in the presence of finite DMI: micromagnetic results

Although the SDM could qualitatively describe the stability phase diagrams, it fails to provide a quantitative description of the experiments^3^,^6^, and the spatial magnetization dependence (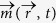) needs to be taken into account for a realistic analysis. Indeed, it has been argued that the Dzyaloshinskii-Moriya interaction (DMI) arises at the interface between the HM (Pt) and the FM (Co) layers^9^,^38^. In particular, it was confirmed that apart from SL-SOT due to the SHE, also the DMI is a key ingredient in governing the statics and dynamics of DWs along ultrathin FM strips sandwiched in asymmetric stacks^9^,^10^,^30^. Similarly to the conventional symmetric exchange interaction (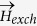) responsible of the ferromagnetic order, the interfacial DMI effective field 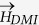 is only different from zero if the magnetization is a non-uniform continuous vectorial function 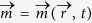. Apart from promoting non-uniform magnetization textures of a definite chirality in the bulk of the FM, the interfacial DMI also imposes specific boundary conditions (DMI-BCs) at the surfaces/edges of the sample^34^. Indeed, for finite DMI (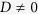), the DMI-BCs ensure that the local magnetization at the edges rotates in a plane containing the edge surface normal 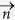, and therefore, in a finite-ferromagnetic dot the uniform state is never a solution, so the SDM does no longer apply. Further details of the 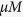 are given in Methods.

### Non-uniform equilibrium states under 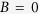 and 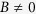 in the absence of current

**Figure 2:**
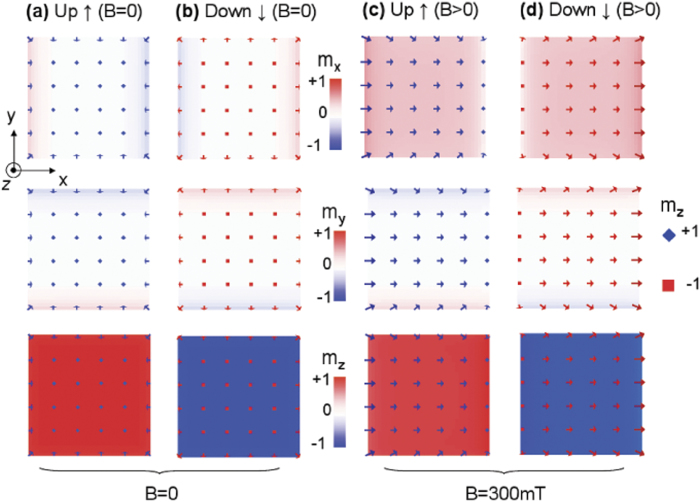
Non-uniform equilibrium magnetization patterns in the presence of finite DMI (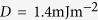) Magnetization snapshots depict the deviations of the local magnetization 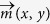 from the perfect out-of-plane direction as due to the DMI-BCs (equation ((7))) at rest (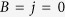) in the presence of interfacial DMI (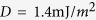) for an state mainly up magnetized (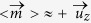, 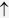) (a), and for an state mainly down magnetized (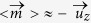, 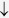) (b). Density plots of the longitudinal 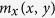, transverse 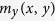 and out-of-plane 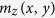 configuration are shown from top to bottom respectively. Arrows show 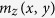. (c) and (d) show the equilibrium state under a positive longitudinal field 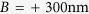 for the 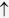 and 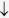 states respectively.

In the equilibrium state at rest (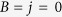), the average magnetization (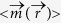, where 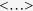 represents the average in the FM volume) points mainly along the easy axis, either along 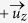 (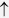, Fig. 2(a)) or 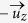 (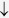, Fig. 2(b)). However, 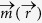 deviates from this easy axis direction at the edges (see Fig. 2). For the up 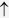 state (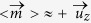), the local magnetization 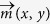 depicts a finite longitudinal component (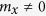), with 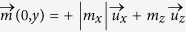 and 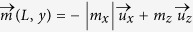 at the left 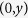 and at the right 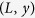 laterals respectively (see Fig. 2(a)). Similarly, 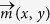 has a non-zero transversal component (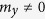), with 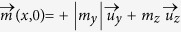 and with 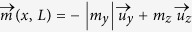 at the bottom 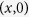 and top 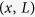 edges respectively. Instead of pointing inwards (Fig. 2(a)), the directions of the in-plane components 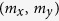 at the edges reverse to outwards for the 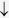 state (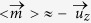, Fig. 2(b)). The deviations from the perfect out-of-plane state are maximum at the edges and decrease over a distance given by 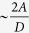 toward to the sample center.

A moderate positive longitudinal field 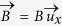 well below the in-plane saturating field slightly modifies the out-of-plane magnetization in the central part of the FM sample, but it introduces significant changes in the local magnetization at the edges, as it can be seen in Fig. 2(c)-(d). A finite longitudinal component 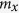 parallel to 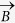 arises at both bottom and top transverse edges 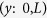 (see 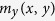 in Fig. 2(c)-(d)). Importantly, the effect of the positive field 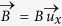 with 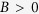 is opposite at the longitudinal left 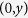 and right 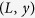 edges. Whereas 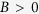 supports the positive longitudinal magnetization component at the left edge 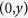, it acts against the negative longitudinal magnetization component at the right edge 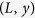 for the 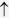 state, as it is clearly seen in Fig. 2(c). For the 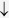 state, 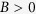 supports the positive 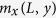 and acts against the negative 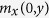 (Fig. 2(d)).

### Non-uniform CIMS from 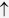 to 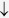 with 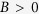 and 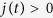 for finite DMI (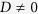)

**Figure 3:**
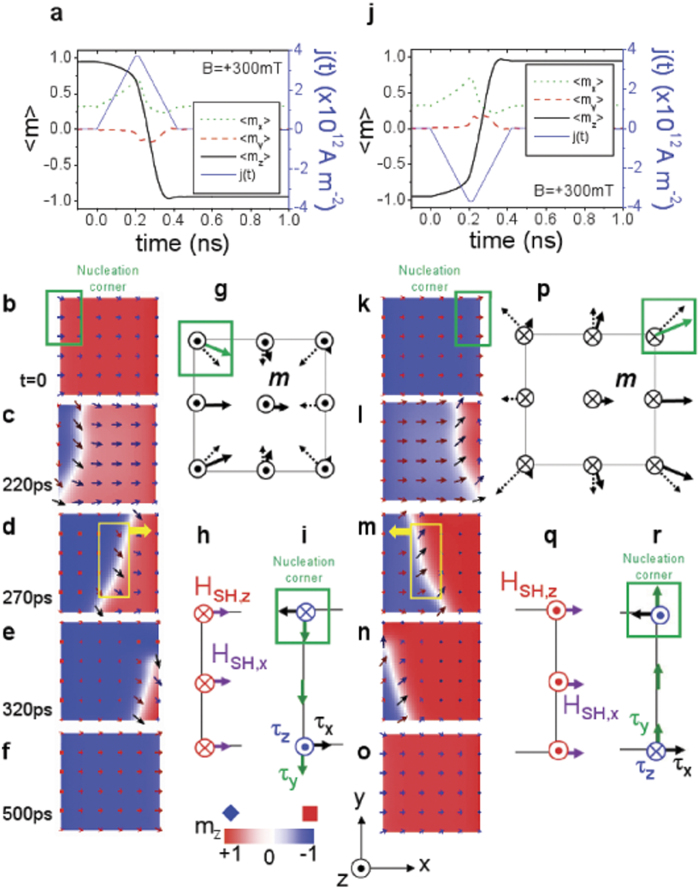
Non-uniform current-induced magnetization switching (CIMS) in the presence of DMI (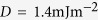) Graphs at the left panel correspond to the 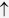 to 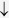 switching under a positive current pulse (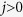). (a) Temporal evolution of the Cartesian components of the magnetization averaged over volume sample (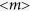) for 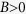 and 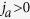. The applied pulse 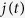 is also shown. (b)-(f) Magnetization 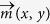 snapshots during the 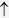 to 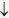 CIMS. Green box in (b) indicates the corner where the switching is triggered as explained in the text and in the schemes (g)-(i): (g) shows 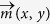 at different points of relevance to understand the CIMS. Dotted arrows indicate the in-plane components of the equilibrium 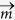 for 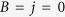, whereas solid vectors indicate the equilibrium state under 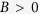. 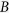 supports the in-plane longitudinal component at the left edge 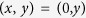. (h) Scheme of the out-of-plane component (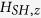, in red) and the in-plane longitudinal component (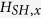, in purple) of the SHE effective field (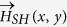) at the left edge corresponding to (b) and (g). (i) Cartesian components of the local torque (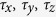) due to 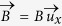 and 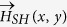 at the relevant left edge: the CIMS is triggered at the top left corner, where 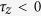 is opposed to the initial out-of-plane up magnetization. Graphs at the right panel (j)-(r) correspond to the 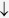 to 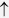 CIMS under 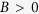 but 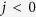. Yellow boxes in (d) and (m) indicate the internal structure of the current-driven domain wall motion due to the SHE.

Since for finite DMI (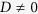) the equilibrium states of Fig. 2 depict non-uniform magnetization patterns 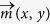, and the SHE effective field depends on the local magnetization (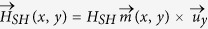), the magnetization dynamics must be also non-uniform, even for the small nano-sized confined dots with 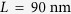 with strong DMI. The non-uniform magnetization dynamics under static longitudinal field (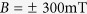) was studied under injection of current pulses 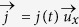 (Fig. 1(b)) with 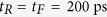, 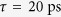 and 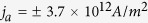 (corresponding to an uniform current 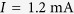 through the Pt/Co section, 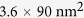) by 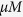 solving the dynamics equation (Methods). The value for the spin Hall angle is 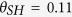 as deduced experimentally by Garello *et al.*^6^ for similar samples. The temporal evolution of the Cartesian magnetization components averaged over the volume of the FM (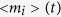 with 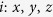) and the current pulse temporal profile (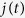) are shown in Fig. 3 for different combinations of 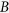 and 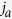 which promote the CIMS from 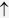 to 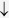 (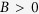 and 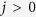), and from 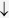 to 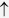 (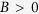 and 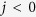). Representative transient magnetization snapshots during the CIMS are also shown in Fig. 3, which clearly indicate that the switching is non-uniform as opposed to SDM predictions.

We focus our attention on the CIMS from 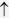 to 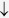 with 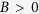 and 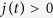 (left graphs in Fig. 3) in the presence of strong DMI (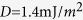). The temporal evolution of the Cartesian magnetization components over the ferromagnet volume (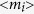 with 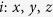) is shown in Fig. 3(a), whereas representative transient magnetization snapshots are shown in Fig. 3(b)-(f). The reversal takes place in two stages. The first one consists on the magnetization reversal at the top left corner of the square resulting in DW nucleation, and the second one occurs via current-driven domain wall (DW) propagation from the left to right due to the SHE. Apart from the snapshots of Fig. 3(b)-(f), these two stages are also evident in the temporal evolution of the out-of-plane magnetization 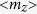 shown in Fig. 3(a). From 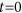 to 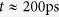, 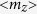 decreases gradually, whereas it decreases almost linearly from 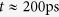 to 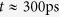, consistent with the current-driven DW propagation where its internal structure is seen in Fig. 3(d).

The magnetization reversal during the first stage is non-uniform due to the DMI imposed boundary conditions (DMI-BCs, see Methods), but to understand in depth the underlaying reasons, it is needed to take into account the chiral-induced non-uniform magnetization (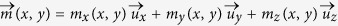) in the presence of the applied field (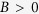) and current (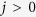). As it can be seen in Fig. 2(c) or in Fig. 3(b), 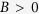 and DMI-BCs support the positive longitudinal magnetization component (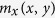) at the left-edge 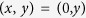, whereas the negative 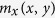 is very small at the right edge 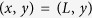. An schematic view of the local equilibrium magnetization at relevant locations is shown in Fig. 3(g) for the 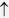 state under 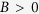 and zero current. The effective SHE field is also non-uniform: 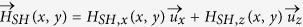 with 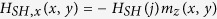 and 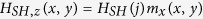. As the out-of-plane component 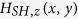 is negative (note that 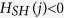 for 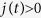) and proportional to the local 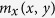, which is maximum and positive at the left edge (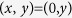), the reversal starts from the left edge (see Fig. 3(h)). However, in addition to this asymmetry along the longitudinal 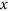-axis imposed by the DMI-BCs and supported by 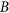 (left 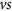 right edges), other chiral asymmetry arises along the transverse 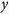-axis in the left edge: the reversal is first triggered from the top left corner (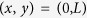), whereas the local CIMS is delayed at the bottom-left corner (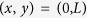), as it clearly seen in Fig. 3(c). The reason for this transverse asymmetry relies in the different direction of local torque at the initial state (Fig. 3(i)). The relevant torque is the one experienced by the local magnetization at the left edge 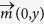 due to 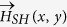, which is also supported by 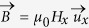: 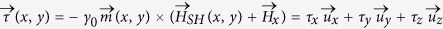. As the local transverse magnetization 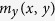 has different sign at the top (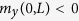) and bottom (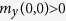) corners of the left edge, both the longitudinal component (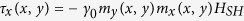) and the out-of-plane component of this torque (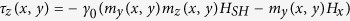) point in opposite directions at the top and the bottom corners of the left edge (see Fig. 3(i)). The relevant component of 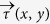 to understand the local reversal is the out-of-plane one: as 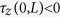 at the top left corner but 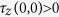 at the bottom left corner, the reversal is firstly triggered from the top corner, where 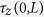 opposes to the initial out-of-plane component of the magnetization (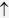). Once the local reversal is achieved at the top left corner, the switching expands from left to right and from top to bottom: the local in-plane magnetization at the bottom left edge rotates clockwise due to 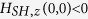, and once 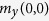 becomes negative, also 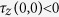 promotes the local reversal.

When all points at the left edge have reversed their initial out-of-plane magnetization (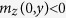) a left-handed (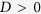) down-up DW emerges, separating the reversed (with 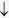) from the non-reversed (with 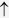) zones. Note that once the local magnetization has reversed its initial out-of-plane direction, it experiences little torque due to 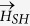 (see Supplementary Information), so it is stable for the rest of the switching process, which takes place by current-driven DW propagation during the second stage.

The internal structure of the propagating DW is shown in Fig. 3(d). Even in the presence of the longitudinal field (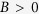), its internal moment (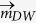) and its normal (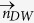) do not point along the positive 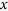-axis, and the DW depicts tilting or a rotation of its normal due to the SHE current-driven propagation. The DW tilting has been experimentally observed in the absence of in-plane field under high currents^39^, and theoretically studied, both in the absence and in the presence of in-plane fields, in elongated strips along the 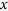-axis^11^,^40^,^41^,^42^. If the only driving force on the down-up DW (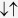) were a strong positive (negative) current 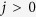 (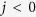) with 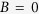, both 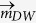 and 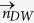 would rotate clockwise (counter-clock wise)^41^. Here, we observe that the DW tilting is also assisted during the DW nucleation due to the DMI-BCs, 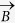 and 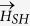. 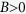 would support the internal longitudinal magnetization of the left-handed down-up DW if its normal points along the 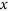-axis (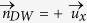), as it would be the case of current-driven DW motion along an elongated strip along the 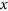-axis^11^. However, due to the non-uniform local CIMS at the left edge in our confined dots, the DW normal has a non-zero negative transverse component (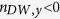) for 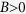 and 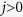. As it is shown in the 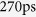-snapshot of Fig. 3(d), in addition to a positive longitudinal component (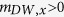), the internal DW moment also has a no-null negative transverse component (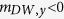). Note that the direction of both 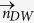 and 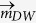 during the DW propagation is also the direction of the local magnetization at the top-left corner, where the reversal was initially launched (see Fig. 3(c),(i)).

The full magnetization switching is completed before the current pulse has been switched off (see Fig. 3(a)), when the propagating down-up DW (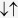) reaches the right edge. Due to the DW tilting, the reversal occurs first at the top right corner (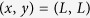) with respect to the bottom right corner (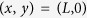) (see 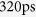-snapshot of Fig. 3(e)). Although this second stage, consisting on current-driven DW propagation, is similar to the one already explained for elongated thin strips as driven by the SHE^11^,^41^,^42^, the DW nucleation during the first stage has not been addressed so far for such small nano-sized confined dots, and as it was explained above it is mainly due to the longitudinal field 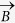 which supports the longitudinal magnetization component at the left edge imposed by the DMI-BCs.

## Discussion

### Universal chiral promoted current-induced magnetization switching (CIMS) in strong DMI systems

The CIMS from 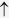 to 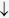 can also be achieved if both 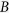 and 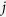 reverse their directions (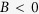 and 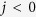). As it is straightforwardly understood from the former description, in this case the reversal is triggered from the bottom right corner (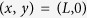, where 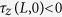 opposes to the initial 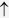 out-of-plane magnetization), and an up-down DW is driven toward the left (not shown). The CIMS from 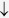 to 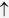 under anti parallel field 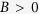 and current 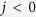 is shown at the right panel of Fig. 3(j)-(r).

In general, the CIMS can be described as follows: (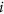) the initial out-of-plane magnetization direction (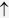 or 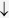) determines the direction (inwards or outwards) of the local in-plane 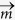 at the edges imposed by the DMI-BCs. (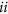) The longitudinal field 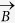 supports the longitudinal in-plane magnetization component (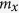) at one of the two lateral edges, and acts against it at the opposite one. (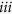) For the favored lateral edge, the local magnetization reversal is triggered at the corner where the out-of-plane torque 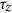 due to 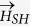 and 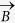 opposes to the initial out-of-plane magnetization component (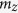). After that, the reversal also takes place in the middle part of the selected edge, and finally, the other corner is also dragged into the reversed region with the formation of a tilted DW. (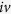) The CIMS is completed by the current-driven DW propagation.

Also remarkable is the fact that for the same current pulses as in Fig. 3 the CIMS is not achieved in the framework of the SDM if a realistic value for the spin Hall angle is adopted (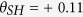)^6^, and the same limitation was also observed by full 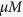 simulations in the absence of the DMI (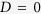). All these simulations point out that, even for the small confined dots considered here (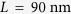), the strong DMI and the BCs imposed by it are essential to describe the CIMS driven by the SHE from both quantitative and qualitative points of view. The DMI-triggered switching (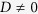) was also studied for other ultrathin (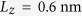) squares (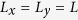) with different in-plane dimensions (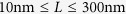) and reversal mechanism remains similar to the one already described and depicted in Fig.3. Note that the smallest evaluated side (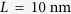) is small than the minimum side required to achieve thermal stability (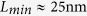) according to the conventional criterion: in order to maintain sufficient stability of the data storage over at least five years, the effective energy barrier given by 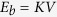 (with 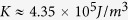 the effective uniaxial anisotropy constant from Ref.^6^, and 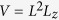 the volume of the sample) should be larger than 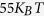, where 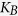 Boltzmann constant. The reversal was also similar under realistic conditions including disorder due to the edge roughness and thermal effects (see Supplementary Information). Moreover, this chiral CIMS, either from 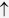 to 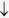 or from 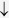 to 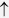, does not change when the FM Co layer is patterned with a disk shape (see Supplementary Information). It was also verified that this non-uniform reversal mechanism, consisting on DW nucleation and propagation, does not depend on the specific temporal profile of the applied pulse, provided its magnitude (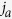) and duration (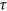) are sufficient to promote the complete reversal for each 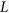.

### Chiral nature of the field-induced magnetization switching (FIMS)

**Figure 4:**
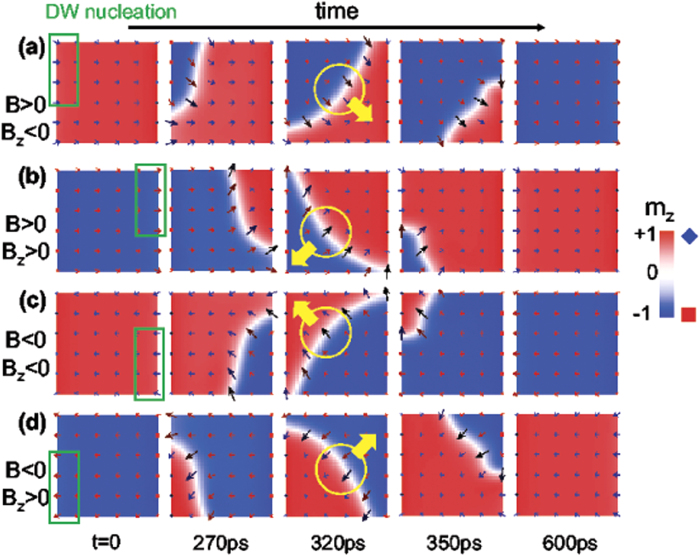
Field-induced magnetization switching in a ultrathin (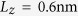) square dot with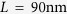 Transient snapshots magnetization 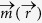 in the presence of interfacial DMI (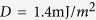) during the reversal for different combinations of 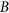 and 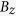 with 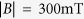, 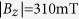, 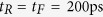 and 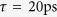. The up (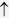) to down (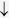) switching is shown for (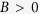,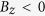) and (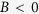,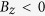) in (a) and (c) panels respectively, whereas the down (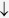) to up (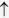) is shown in (b) and (d) for (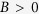, 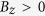) and (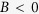,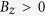). Green boxes indicate the region where the DW nucleation starts for each combination of initial state (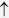 or 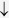), 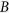 and 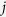. Yellow circles indicate the field-driven propagating DW (yellow arrow indicate the direction of the reversed domain expansion). Note that the in-plane components of the nucleation region (green box) point along close to the internal DW moment (yellow circle) during its propagation.

An analogous CIMS mechanism to the one described here for nano-size samples (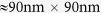) was recently observed by Yu *et al.*^43^ using Kerr microscopy for an extended Ta(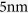)/CoFeB(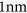)/TaO(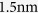) stack with micro-size in-plane dimensions (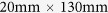). In that work, right-handed DWs (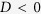) were nucleated assisted by the in-plane field and displaced along the current direction due to the negative spin Hall angle of the Ta. More recently, Pizzini *et al.*^38^ also used Kerr microscopy to visualize the asymmetric chiral DW nucleation under in-plane field and its subsequent propagation along extended (≈70 μm) Pt(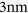)/Co(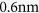)/AlO(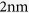) thin-films driven by out-of-plane field (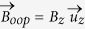). Similar to our study, starting from the up state (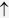), a positive (negative) in-plane field (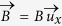) promotes the local magnetization reversal at the left (right) edge, which was propagated to the right (left) driven by a negative out-of-plane field 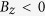. Their images indicate the nucleated DW has a left-handed chirality and it propagates without significant tilting due to the extended unconfined in-plane dimensions (≈70 μm). In order to understand these observations, the field-induced magnetization switching (FIMS) has been also studied for confined small squares with 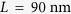 (the same geometry as in the former CIMS analysis) and others with lateral dimensions one order of magnitude larger (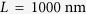). Static longitudinal fields 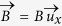 with 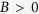 and 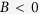 (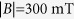) are applied along with short out-of-plane field pulses with 
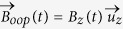
 with 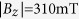, 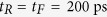 and 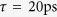 (the temporal profile of this pulse is the same as for the current-induced magnetization switching). The results for the confined 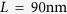 square dot are shown in Fig 4 for different combinations of the initial state (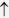 and 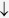), in-plane static field 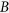 (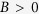 and 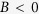) and out-of-plane field pulse (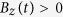 and 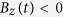). Similarly to the CIMS, the FIMS starts from an edge selected by the direction of 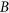, with an even more evident chiral asymmetry between the two corners. Note again that the corner where the reversal starts has a transverse magnetization component (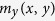) pointing in the same direction as the transverse internal magnetization of the nucleated DW. Once the local switching has been triggered, the reverse domain (pointing along the opposite 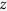 direction with respect to the initial state) expands asymmetrically along the longitudinal (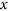) and transverse (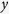) directions (see for instance snapshots at 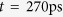 and 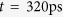 in Fig. 4). Although here just a quarter-of-bubble is developed due to the confined shape at the corner, this asymmetric field-driven chiral expansion is similar to the one recently observed^12^,^14^ in extended thin films. Moreover, our study also points out a qualitative difference between the current-driven and the field-driven nucleation: while the first one is driven by a non-uniform SHE out-of-plane effective field (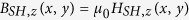 which depends on local 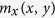), the second one is promoted by a uniform out-of-plane field 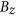. Therefore, the current-induced nucleated DW propagates along the current direction (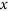-axis, see yellow arrows in Fig. 3(d) and (m)), whereas the field-driven DW expands radially from the corner (see yellow arrows in Fig. 4). Nevertheless, the fact that similar chiral local magnetization reversal occurs also at the corners of nano-size confined dots (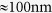 and below) clearly confirms the universality of the chiral reversal mechanism in these nano-size confined dots with strong DMI.

On the other hand, the Kerr images by Pizzini *et al.*^38^ do not show the corners of their extended thin-film (which is unconfined along the transverse 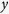-axis) which are precisely where our modeling points out additional chiral asymmetry in the DW nucleation for confined dots (Fig. 4). Moreover, in their thin-films the field-driven DW does not depict tilting. In order to contrast these observations with our 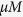 predictions, the field-driven nucleation and propagation in an confined square dot has been also analyzed here, but with lateral in-plane dimensions one order of magnitude larger (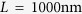). We note that as 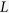 is increased to the microscale, the nucleated DW is almost straight, with its normal oriented along the 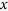-axis (no DW tilting), in the middle part of the nucleating edge (far form the corners). However, an asymmetry between the top and bottom corners is still present even for 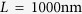 (see Supplementary Information): the reversal from 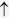 to 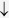 (from 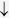 to 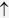) is anticipated at the top-left (top-right) corner with respect to the bottom one under 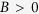 and 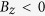 (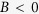 and 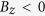). This chiral asymmetry at the corners of the extended micro-size sample is similar to the observed for a confined dot (see. Fig. 4), and although it has not been addressed before, it could be observed by high resolution techniques^44^.

### CIMS in confined nanodots with rectangular shape

**Figure 5:**
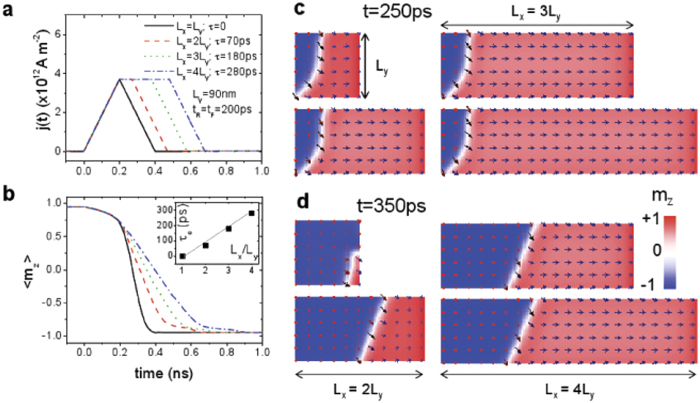
Current-induced magnetization switching along thin rectangles CIMS in rectangles with 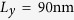 and different aspect-ratio 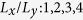. The applied field 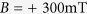 and the current pulses have 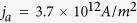 and 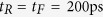 fixed, and different durations 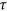 depending on 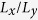 (a). (b) Temporal evolution of the out-of-plane component 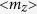 for different rectangles under the pulses shown in (b). Snapshots of the magnetization state at 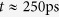 (c) (DW nucleation) and at 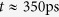 (d). The inset in (a) shows the critical threshold for 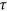 as a function of the 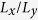.

The CIMS was also studied in rectangles with different in-plane aspect-ratios 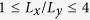 (Fig. 5(a)-(d)). The thickness is fixed (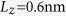) as before. Again the switching takes place by DW nucleation followed by its current-driven propagation along the 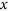-axis, which further supports the universality of the reversal mechanism in systems with strong DMI. In this case, the nucleation takes place during the first 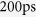 independently of the rectangle aspect-ratio 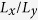, but the critical pulse duration (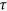) for fixed 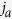 and 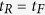, increases linearly with 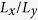 (see the inset in Fig. 5(c)), a prediction which could be experimentally validated to estimate both the spin Hall angle (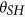) and the DMI parameter (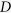) if the rest of material parameters (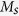, 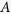, 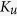, 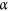) are known by other means.

### Comparison to experiments of current-induced magnetization switching

**Figure 6:**
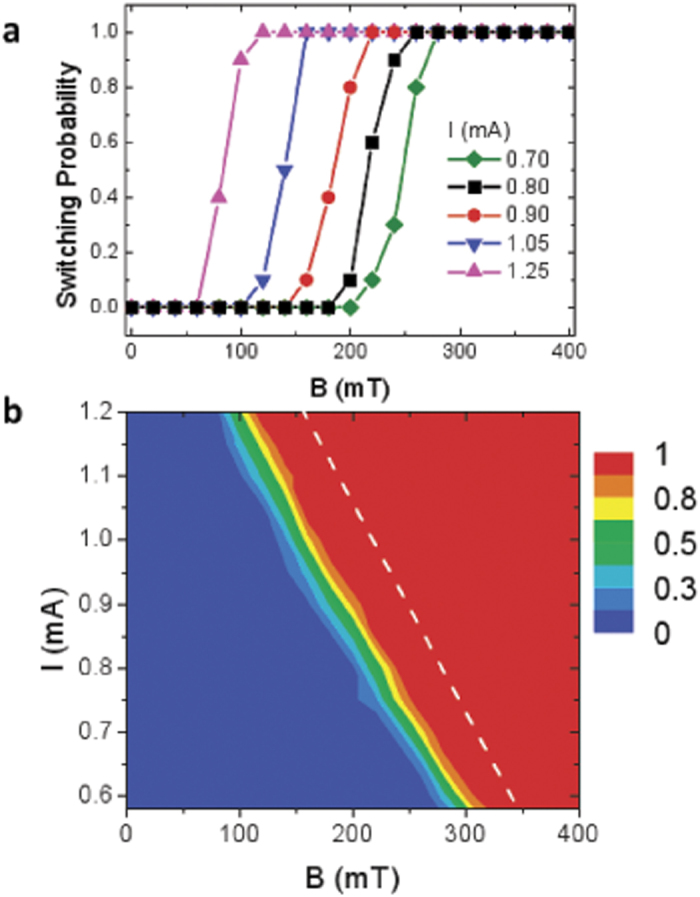
Quantitative description of experimental results Micromagnetically computed switching probability as function of the applied field 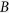 and the current pulse 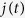. Similar current pulses as in the experiments by Garello *et al.*^6^ are applied: 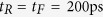 and 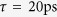 are fixed, and different magnitudes 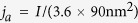 are studied (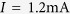 corresponds to 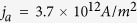). Results were computed at room temperature 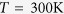 by averaging over 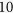 stochastic realizations. (a) Switching probability as a function of 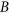 for pulses with several magnitudes expressed in term of 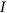 as in the experimental study. The switching probability as function 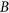 and 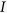 is depicted by density plot in (b). Dashed white curve in (b) represents the threshold between not-switching and switching computed at zero temperature.

Although our study goes further than a mere comparison to available experimental results, it is interesting to show how the non-uniform CIMS can explain quantitatively the experimental measurements by considering realistic material parameters (see Methods and Supplementary Information). With the aim of providing an explanation of experimental observations^6^ for the ultrahin Co square with 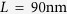 in a Pt(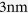)/Co(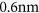)/AlO(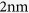) stack, we have repeated the former study for several values of the applied field (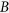) and different different magnitudes of the current pulse (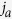). The rise and fall times (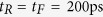) and the duration (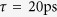) of the pulse were maintained fixed as in the experimental study^6^. Here we consider the up state (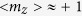, 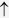) as the initial one. For each 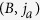, the switching probability at room temperature was computed as the averaged over 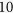 stochastic realizations. Realistic conditions were taken into account by considering random edge roughness with characteristic sizes ranging from 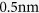-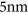 (see Methods). The 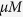 results are collected in Fig. 6 which indicates a good quantitative agreement with recent experimental measurements^6^.

It was verified that the CIMS mechanism (local magnetization reversal with DW nucleation and subsequent current-driven propagation) remains qualitatively unchanged even under these realistic conditions (see Supplementary Information). Moreover, although marginal discrepancies between these 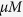 data (Fig. 6) and the experimental results shown in Fig. 2(d) of ref.^6^ can be seen, the quantitative agreement is remarkable considering similar material parameters as inferred experimentally^6^: 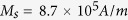, 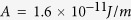, 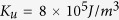, 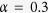, 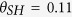 and 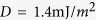 (see Supplementary Information for detailed justification of these inputs). Note that with the SDM a quantitative agreement with the experimental data was only achieved with unrealistic values of the (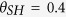)^6^. Note that the DMI parameter 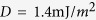 was not determined experimentally^6^, but the fact that this value 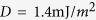 provides reasonable quantitative agreement with their experiments, and that this value is also in good quantitative agreement with very recent estimations by other means for similar Pt-based systems (Ref. 11) constitute additional evidences that our modeling is compatible with the dominant physics behind these CIMS processes.

## Conclusions

In summary, the current-driven magnetization switching in ultrathin HM/FM/Oxide heterostructures with high PMA and strong DMI has been studied by means of full micromagnetic simulations. Even for the small in-plane dimensions (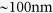), the analysis points out that the magnetization reversal mechanism is non-uniform. It starts by local magnetization reversal induced by the SHE and assisted by the in-plane field in collaboration with the DMI boundary conditions. The longitudinal field and the DMI imposed boundary conditions select the lateral/edge and the specific corner at which the nucleation is triggered, where the relevant torques due to the SHE and the longitudinal field accelerate the local reversal. After that, the switching is completed by current-driven domain wall propagation driven by the SHE, where the current direction determines the direction of the wall motion, and the internal magnetization of the propagating wall points closely to the local magnetization at the selected corner where the reversal was initially launched. Similar nucleation and propagation mechanisms were also observed under out-of-plane fields, confirming again the chiral-triggered magnetization reversal in these nano-size confined dots. These results clearly exclude the single domain approach as a proper model to describe these switching experiments, and therefore, the estimations of the spin Hall angle based in this oversimplified model should be revised by adopting a much more realistic full 3D micromagnetic approach. Moreover, by analyzing the switching under realistic conditions including disorder and thermal effects, it was found that the mechanism is universal, and for instance, it could be used to the quantify both the DMI and the spin Hall angle by studying the reversal of ferromagnetic layers with different length for fixed width and thickness. As the reversal mechanism occurs in a reliable and efficient way, and more importantly, as it is also highly insensitive to defects and thermal fluctuations, our results are also very relevant for technological recording applications combining non-volatility, high stability, ultra-dense storage and ultrafast writing.

## Methods

### Magnetization dynamics under SOT due to the SHE

Under injection of a spatially uniform current density pulse along the 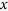-axis 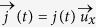 (see its temporal profile in Fig. 1(b)), the magnetization dynamics is governed by the augmented Landau-Lifshitz Gilbert eq.


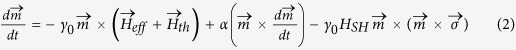


where 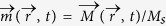 is the normalized local magnetization with 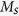 saturation magnetization, 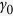 is the gyromagnetic ratio and 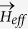 is the effective field derived from the energy density of the system (
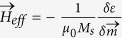
). The first term in equation (2) represents the precessional torque of 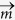 around 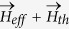, where 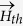 is the thermal field representing the effect of thermal fluctuations at finite temperature. 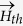 is a white-noise Gaussian-distributed stochastic random process with zero mean value (its statistical properties are given below). The second term in equation (2) is the damping torque with 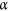 the dimensionless Gilbert damping parameter. The last term in equation (2) is the SL-SOT from the spin Hall effect (SHE), where 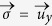 is the unit vector pointing along the direction of spin current polarization due to the SHE in the Pt layer, and 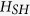 represents the magnitude of the effective spin Hall field 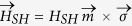 given by


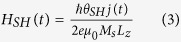


where 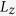 is thickness of the FM layer, 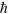 is Planck’s constant, 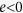 is the electron charge and 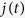 is the instantaneous value of the electrical density current. As in the experiment by Garello *et al.*^6^, the current is assumed to flow uniformly through the HM/FM bilayer (see Supplementary Information for additional discussion). 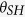 is the Spin Hall angle, which is defined as the ratio between the spin and charge current densities.

### Single Domain Model (SDM)

If the magnetization is assumed to be spatially uniform (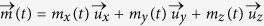), the deterministic effective 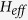 field in equation (2) only includes the PMA anisotropy, magnetostatic and Zeeman contributions 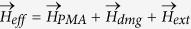. The Zeeman contribution due to the longitudinal field is 
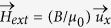
. The uniaxial PMA anisotropy effective field is


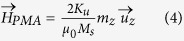


and the demagnetizing field in the SDM approach is expressed as


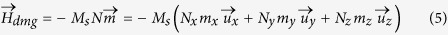


where 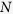 is the diagonal magnetostatic tensor with 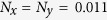 and 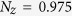 being the self-magnetostatic factors^45^ for 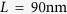 and 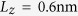.

The thermal field 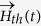 is a stochastic vector process whose magnitude is related to the temperature 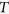 via the fluctuation-dissipation theorem^46^.


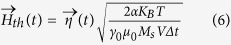


where 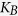 is the Boltzmann constant, 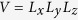 is the volume of the sample, 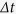 is the time step, and 
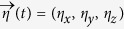
 is a Gaussian distributed white-noise stochastic vector with zero mean value (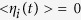 for 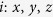) and uncorrelated in time (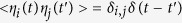, where 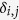 is the Kronecker delta and 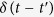 the Dirac delta). Here 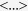 means the statistical average over different stochastic realizations of the stochastic process. Equation (2) was numerically solved with a 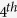-order Runge-Kutta scheme with a time step of 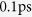.

### Micromagnetic Model (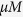)

When the spatial dependence of the magnetization is taken into account (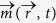), the deterministic effective field 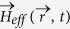 in equation (2) includes the space-dependent exchange 
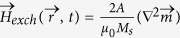
 with 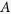 the exchange constant, and the interfacial DMI 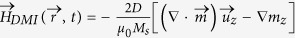
30,34 where 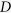 is a parameter describing the DMI magnitude. Both the local Zeeman and PMA uniaxial contributions to 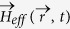 are computed similarly as in the SDM (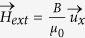 and 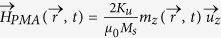). Note also that in the 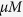 the magnetostatic field 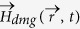 is also space-dependent on 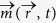 everywhere. The Oersted field due to the current was also taken into account but it was found irrelevant and very small as compared to the other dominant contributions in 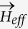. (see^47^,^48^ for the numerical details).

In the absence of DMI (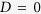), the symmetric exchange interaction imposes boundary conditions (BCs) at the surfaces of the sample^49^ so that 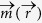 does not change along the surface (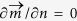, where 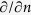 indicates the derivative in the outside direction normal to the surface of the sample). However, in the presence of the interfacial DMI (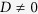), these BCs have to be replaced by^11^,^34^


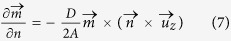


where 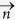 represents the local unit vector normal to each sample surface.

In the 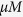 the thermal field 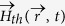 is also a stochastic vector process given by


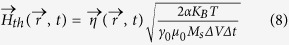


where now 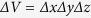 is the volume of each computational cell and 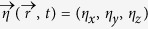 is a white-noise Gaussian distributed stochastic vector with zero mean value (
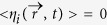
 for 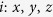) and uncorrelated both in time and in space (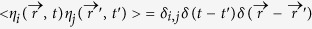). Most of the simulations for perfect samples were performed with a 2D discretization using cells of 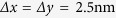 in side, and thickness equal to the ferromagnetic layer (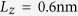). Several tests were performed with cell sizes of 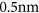 to confirm the numerical validity of the presented results. Realistic samples were also studied by considering edge roughness using cell sizes of 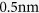. These realistic conditions are introduced by randomly generating edge roughness patterns with different characteristic sizes 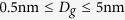 at all edges. Equation (2) was numerically solved with a 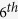-order Runge-Kutta scheme with a time step of 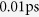 by using GPMagnet^47^, a commercial parallelized finite-difference micromagnetic solver^48^.

### Material parameters

Typical high PMA material parameters were adopted for the results collected in the main text in agreement with experimental values for Pt/Co/AlO^5^,^6^,^38^: saturation magnetization 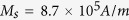, exchange constant 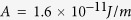, uniaxial anisotropy constant 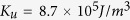. The spin Hall angle is assumed to be 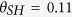, also according to experiments by Garello *et al.*^5^,^6^. Note that this value is also in the middle of the experimental bounds 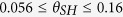 estimated by Liu *et al.*^2^ and Garello *et al.*^5^, and very close to the one deduced in^13^. A DMI parameter of 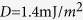 is assumed, which is similar to the one experimentally deduced by Emori *et al.*^11^. The Gilbert damping is 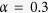 as measured in^50^. Several tests were also performed by varying these inputs within the range available in the experimental literature (see Supplementary Information).

## Additional Information

**How to cite this article**: Martinez, E. *et al*. Universal chiral-triggered magnetization switching in confined nanodots. *Sci. Rep.* **5**, 10156; doi: 10.1038/srep10156 (2015).

## Supplementary Material

Supplementary Information
