# Supplementary material for: Universal chiral-triggered magnetization switching in confined nanodots
Source: Sci Rep. 2015 Jun 10;5:10156. doi: 10.1038/srep10156 (PMC4650651; doi:10.1038/srep10156)
Supplement: Supplementary Information [file srep10156-s1.pdf]

Supplementary Information: Universal chiral-triggered magnetization switching in confined nanodots.

Eduardo Martinez[1] *, Luis Torres[1], Noel Perez[1], Maria Auxiliadora Hernandez[1], Victor Raposo[1] and Simone Moretti[1]

[1] *Universidad de Salamanca. Plaza de los Caidos s/n, E-37008, Salamanca. Spain.*

(Dated: February 28, 2015)

* Corresponding author's e-mail: edumartinez@usal.es

I. CURRENT-INDUCED MAGNETIZATION SWITCHING (CIMS): SPIN HALL EFFECTIVE FIELD

Here we repeat the current-induced magnetization switching of the ultrathin square ($L =$ 90nm, $L_z = 0.6$nm) from the up state ($\uparrow$) under $B = 300$mT and a current pulse with $j_a = 3.7 \times 10^{12}$A/m^2, $t_R = t_F = 200$ps and $\tau = 20$ps as discussed and depicted in Fig. 3a of the main. In particular, here we present the local out-of-plane effective spin Hall field $H_{SH,z}(\vec{r})$ during the switching process. The results are shown in Fig. S. 1.

As it can be clearly seen, the normalized $h_{SH,z}(\vec{r}) = H_{SH,z}(\vec{r})/|\vec{H}_{SH,z}(\vec{r})|$ is maximum at the left edge and points along the negative z-axis during the DW nucleation (Fig. S.1(b), $t = 209$ps). During the subsequent DW propagation (Fig. S.1(c)-(f)), $h_{SH,z}(\vec{r})$ is close to zero at the reversed areas (domain at the left side of the nucleated DW) and very small at the still no-reversed domain (domain right side of the DW). More importantly, the maximum values of $h_{SH,z}(\vec{r})$ are precisely inside the DW, confirming again that it is the driving force on wall.

II. CURRENT-INDUCED MAGNETIZATION SWITCHING (CIMS) UNDER REALISTIC CONDITIONS

The current-induced magnetization switching of the ultrathin square ($L = 90$nm, $L_z = 0.6$nm) was also analyzed under realistic conditions considering edge roughness (with characteristic grain sizes D_g from 0.5nm to 5nm) and thermal effects ($T = 300$K). Typical results are shown in Fig. S.2 for the same field and current pulse as in the Fig. 3(a)-(f) of the main text. The reversal mechanism remains insensitive to these effects.

III. THE ROLE OF CONVENTIONAL SPIN TRANSFER TORQUES (STTS) IN THE CIMS

Results presented in the main text were computed by assuming negligible spin polarization of the charge current in the ferromagnet ($P = 0$), because it is expected to be vanishingly small as the thickness of the sample is reduced to atomistic size, as reported in experimental studies[9,13–15]. Here we show that even in the case of unrealistically high

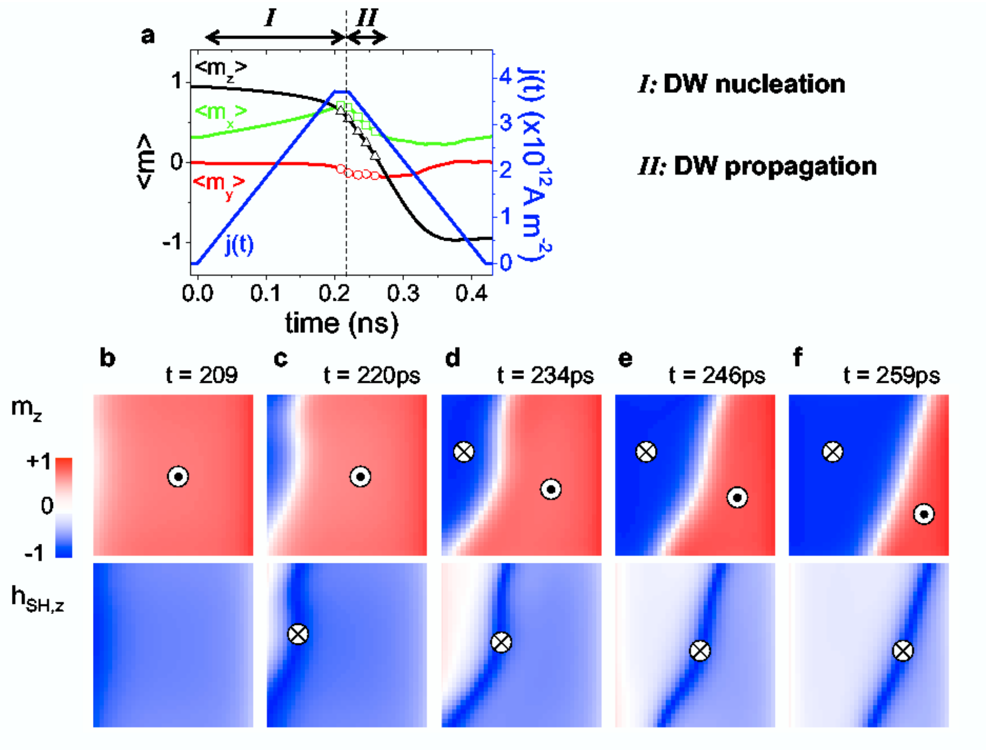

FIG. 1: (Color online) Details of the CIMS of Fig. 3a of the main text. (a) temporal evolution of averaged magnetization components and the current pulse. (b)-(f) show transient snapshots of the out-of-plane component of the magnetization ($m_z(\vec{r})$, top graphs) and the normalized out-of-plane component of the spin Hall effective field ($h_{SH,z}(\vec{r})$, bottom graphs).

spin polarization ($P = 0.5$), the conventional adiabatic and non-adiabatic spin transfer torques (STT) play a negligible role in the current induced magnetization switching. The current-induced magnetization switching of the ultrathin square ($L = 90$nm, $L_z = 0.6$nm) was also analyzed considering the influence of the conventional adiabatic and non-adiabatic spin transfer torques (STT)[12,22]. The results are shown in Fig. S.3. In the absence of SHE ($\theta_{SH} = 0$), these STTs can not drive the switching by themselves. In the presence of SHE ($\theta_{SH} = 0.11$), the STTs only slightly delay the reversal but do not modify the reversal mechanism.

IV. CURRENT INDUCED MAGNETIZATION SWITCHING IN THIN DISK

Similar CIMS reversal mechanism was also observed for a thin disk with diameter 90nm, the same as the side of the square of the main text. The thickness is also the same ($L_z =$

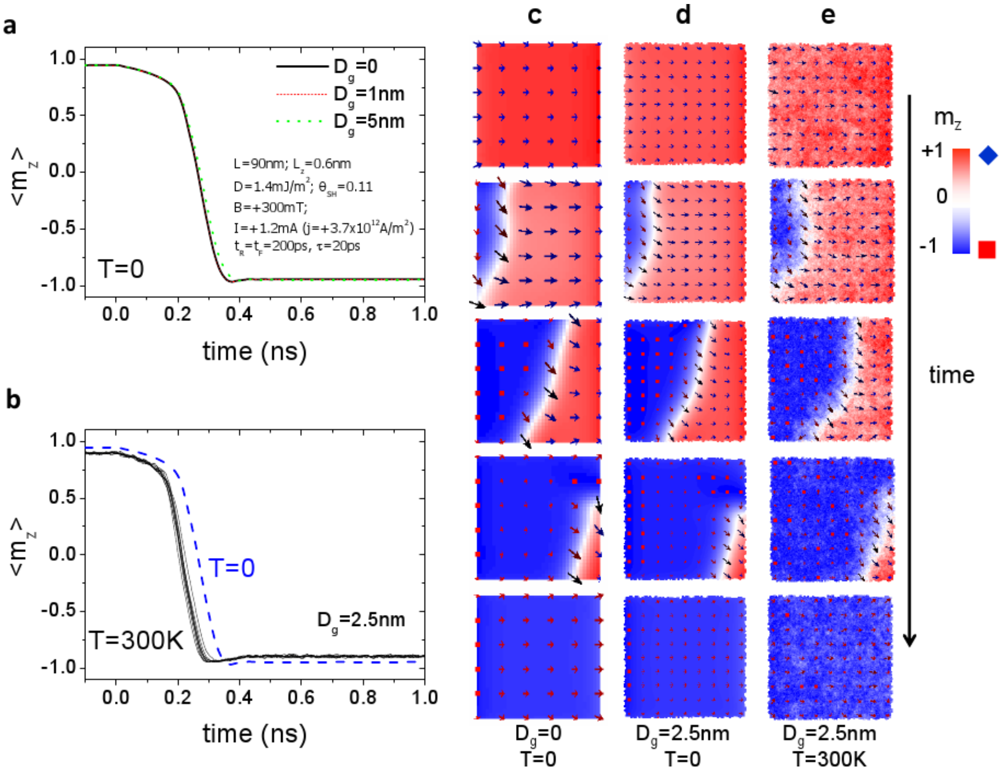

FIG. 2: (Color online) Current-induced magnetization switching under realistic conditions. The same dimensions, field and current pulses as in Fig. 3(a)-(f) of the main text are considered: $B = +300\text{mT}$, $j_a = 3.7 \times 10^{12}\text{A/m}^2$, $t_R = t_F = 200\text{ps}$ and $\tau = 20\text{ps}$. The up ($\uparrow$) to down ($\downarrow$) switching is shown for $B > 0$ and $j > 0$. The temporal evolution of the out-of-plane magnetization averaged over the ferromagnet volume are shown in (a) for a perfect sample an others with different edge roughness (D_g) at $T = 0$. (b) m_z *vs* time for $D_g = 2.5\text{nm}$ at $T = 0$ (dashed blue line) and ten stochatic realizations at $T = 300\text{K}$ (thin black lines). Corresponding magnetization snapshots are also displayed from comparison in (c) $D_g = 0$, $T = 0$, (d) $D_g = 2.5\text{nm}$, $T = 0$, and (e) $D_g = 2.5\text{nm}$, $T = 300\text{K}$.

0.6nm). The magnetization snapshots during the reversal for different combinations of B and j are shown in Fig. S.4. These simulations also show the chiral asymmetry during the reversal, qualitatively similar to the results obtained for squares.

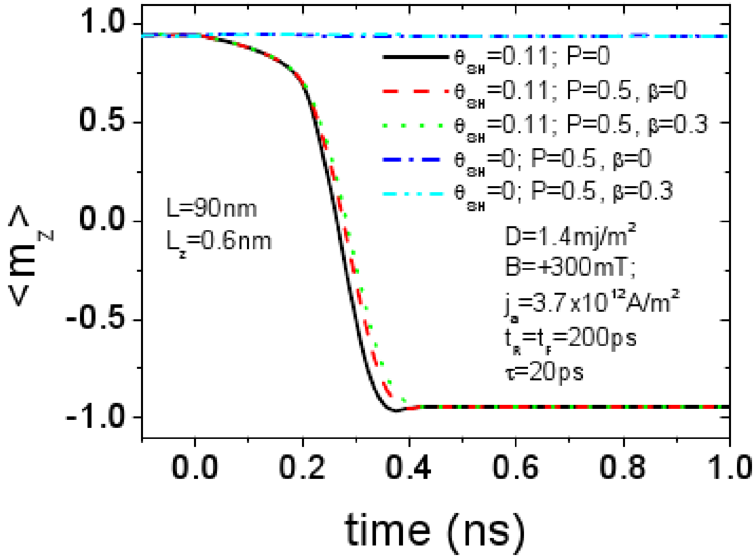

FIG. 3: (Color online) The influence of the conventional STTs on the current-induced magnetization switching of the ultrathin square (L = 90nm, L_z = 0.6nm). The spin polarization was assumed to be $P = 0.5$ and both perfect adiabatic ($\beta = 0$) and non-adiabatic ($\beta = 0.3$) conditions are analyzed. The reversal is only achieved in the presence of the SHE ($\theta_{SH} = 0.11$), and the conventional STTs slightly delay the reversal without modifying the reversal mechanism. The material parameters, the in-plane longitudinal field and the current pulse are the same as in former Fig. S.1 and Fig. 3 of the main text.

V. FIELD-INDUCED MAGNETIZATION SWITCHING (FIMS) IN EXTENDED MICRO-SIZE SQUARE

The field-induced magnetization switching was studied for an extended L = 1000nm square dot with the same thickness 0.6nm as the small nanodot square of the main text. The field-driven magnetization reversal is studied under a static longitudinal in-plane field $\vec{B} = B\vec{u}_x$ with B = 300mT and a time-varying out-of-plane field $\vec{B}_{oop}(t) = B_z(t)\vec{u}_z$ increasing linearly from 0 to $|B_z|$ = 310mT with a rising time of t_R = 200ps, and after that maintained constant until the end of the simulations. The snapshots during the reversal for different combinations of B and B_z are shown in Fig. S.5. This analysis indicates that far from the corners (in the middle part of the nucleation edge) the nucleated domain wall is almost straight, with normal oriented along the x-axis (no DW tilting). However, the asymmetry is still evident between the top and the bottom corners: the reversal from $\uparrow$ to $\downarrow$ (from $\downarrow$

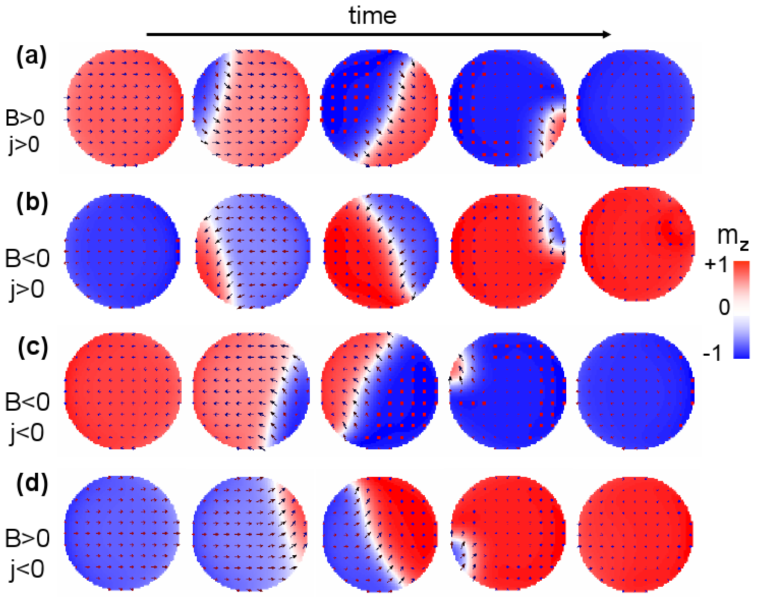

FIG. 4: (Color online) Transient snapshots magnetization $\vec{m}(\vec{r},t)$ in the presence of interfacial DMI ($D = 1.4\text{mJ/m}^2$) during the CIMS of 0.6nm-thick Co disk with diameter 90nm. Different combinations of B and j with $|B| = 300\text{mT}$, $|j_a| = 3.7 \times 10^{12}\text{A/m}^2$, $t_R = t_F = 200\text{ps}$ and $\tau = 20\text{ps}$ are evaluated. The up (↑) to down (↓) switching is shown for ($B > 0$,$j > 0$) and ($B < 0$,$j < 0$) in (a) and (c) panels respectively, whereas the down (↓) to up (↑) is shown in (b) and (d) for ($B < 0$,$j > 0$) and ($B > 0$,$j < 0$).

to ↑) is anticipated at the top left (top-right) corner with respect to the bottom one under $B > 0$ and $B_z < 0$ (under $B < 0$ and $B_z < 0$).

VI. CURRENT DISTRIBUTION AND OERSTED FIELD

A. Assuming the same conductivity in the heavy-metal and the ferromagnet

The results shown in the main text were computed by assuming that the current flows uniformly distributed along Pt/Co layers, so the effective conducting thickness is 3.6nm. This is the common assumption in the experimental studies to estimate the spin Hall angle[3,7], in particular, in the experimental work by Garello et al.[5] that we have reproduced, not only qualitatively but also quantitatively.

The electrical current distribution in the Pt/co bilayer was numerically computed by means of COMSOL[24] simulations assuming that the Co has the same resistivity as the Pt:

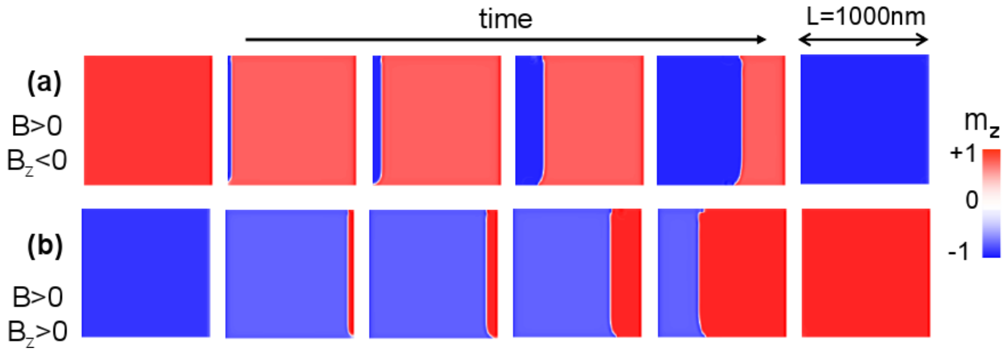

FIG. 5: (Color online) Transient snapshots magnetization $\vec{m}(\vec{r})$ in the presence of interfacial DMI ($D = 1.4\text{mJ/m}^2$) during the field-driven switching of a Co square with $L = 1000\text{nm}$ and $L_z = 0.6\text{nm}$. Different combinations of B and B_z with $B = +300\text{mT}$, $|B_z| = 310\text{mT}$ with a rising time $t_R = 200\text{ps}$. The up ($\uparrow$) to down ($\downarrow$) switching is shown in (a) for ($B > 0$,$B_z < 0$), whereas the ($\downarrow$) to ($\uparrow$) reversal is shown (b) in with ($B > 0$,$B_z > 0$).

$\rho(Co) = \rho(Pt) = 105 \times 10^{-9}\Omega\text{m}$. The spacial distribution of the current is shown in Fig. S.6 when a $j_a = 10^{12}\text{A/m}^2$ is injected in the Pt layer ($I(Pt) = j_a S_{Pt} = 0.27\text{mA}$, where $S_{Pt} = 90 \times 3\text{nm}^2$ is the cross section of the Pt layer). When this current reaches the area of the Pt/Co bilayer it distributes uniformly across both layers as shown in Fig. S.6(c). A simple estimation based on the Ohm's law indicates that 20% of the total current would flow through the Co layer, in good quantitative agreement with the results shown in Fig. S.6(c). Such small current in the Co layer and the fact that it has negligible bulk spin polarization for its atomic thickness, further support the marginal influence of conventional STTs in the analysis.

The Oersted field ($\vec{B}_{Oe}$) could play also a role in the DW nucleation and current-driven dynamics. However, a COMSOL analysis[24] considering the applied current and its spatial distribution clearly indicates that its effect is indeed negligible. The Oersted field $\vec{B}_{Oe}(\vec{r})$ generated by the current distribution in the Pt/Co bilayer is shown in Fig. S.7. $\vec{B}_{Oe}$ in the Co layer reaches its highest magnitude at top ($y = L$) and bottom ($y = 0$) edges where $|B_{Oe,max}| \approx 3\text{mT}$ for $j_a = 10^{12}\text{A/m}^2$ (see Fig. S. 7(a)-(b)), which would result in $|B_{Oe,max}| \approx 12\text{mT}$ for $j_a = 4 \times 10^{12}\text{A/m}^2$. This value is ≈ 20 times smaller than the in-plane and SHE effective fields, so it does not modify significantly the results, as it was confirmed by full micromagnetic simulations including it for several tested cases.

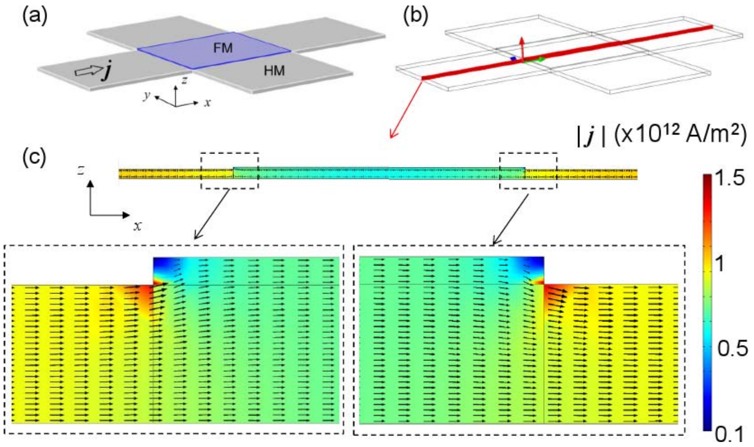

FIG. 6: (Color online) Current distribution computed by COMSOL[24] in the studied sample considering that both the FM Co and the HM Pt layers have the same resistivities: $\rho(Co) = \rho(Pt) = 105 \times 10^{-9}\Omega$m, which is the resistivity of the Pt at room temperature. The injected current density through the Pt far from the Co layer is $j_a = 10^{12}$A/m^2.

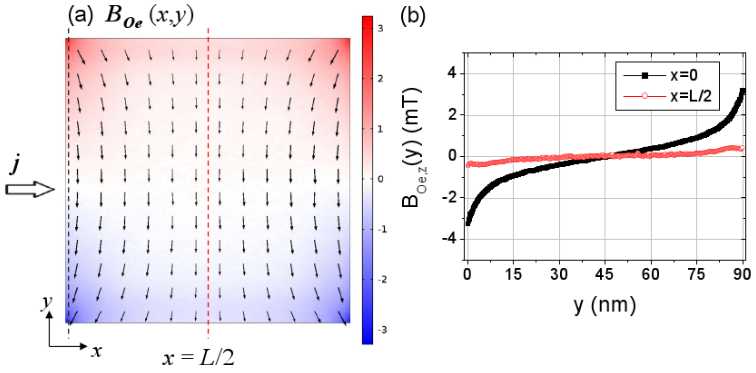

FIG. 7: (Color online) Oersted field $\vec{B}_{Oe}$ in the Co layer computed by COMSOL[24] corresponding to Fig. S.6. The injected current density through the Pt far from the Co layer is $j_a = 10^{12}$A/m^2.

B. Considering the different conductivities in the heavy-metal and the ferromagnet

As it was mentioned above, for our quantitative description of the experiments by Garello[5] we have assumed that the current flows uniformly distributed along Pt/Co layers, which is also the conventional assumption in most of the experimental studies to estimate the spin Hall angle[3,5,7]. Here we point out that a more precise analysis would need to be adopted accounting for the different electrical resistivity of the Co and the Pt: $\rho(Co) =$

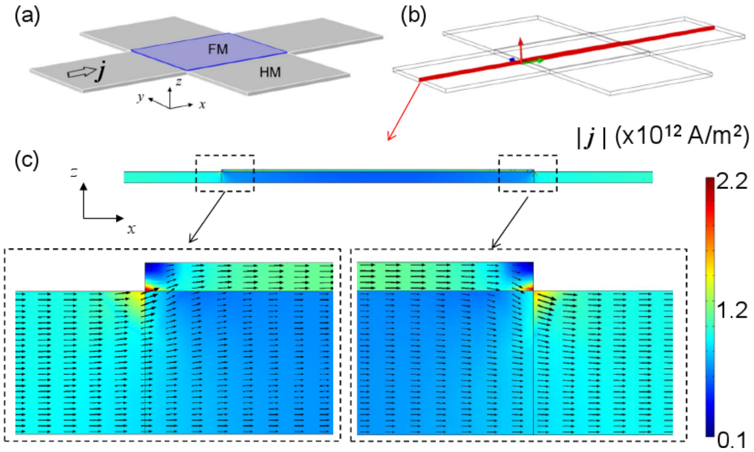

FIG. 8: (Color online) Current distribution computed by COMSOL[24] in the studied sample considering the different electrical resistivity of the Co and the Pt: $\rho(Co) = 62.4 \times 10^{-9}\Omega$m for Co, whereas $\rho(Pt) = 105 \times 10^{-9}\Omega$m for Pt, both at room temperature. The injected current density through the Pt far from the Co layer is $j_a = 10^{12}$A/m^2.

$62.4 \times 10^{-9}\Omega$m for Co, whereas $\rho(Pt) = 105 \times 10^{-9}\Omega$m for Pt, both at room temperature. Therefore, in the studied stack the current density is not uniformly flowing in the Pt/Co bilayer, and a simple estimation confirmed by COMSOL simulations[24] (see Fig. S.8) clearly indicates that even for the small Co thickness (0.6nm) as compared to the Pt layer (3nm), the 36% of the current would flow through the Co layer. This non-uniform distribution of the current would reduce the electrical current in the Pt, and consequently the SHE efficiency H_{SH}/j which is determined by the spin Hall angle $H_{SH}/j \propto \theta_{SH}$. Therefore, as the current flows differently through the Pt and Co, the experimental estimations of the θ_{SH} should be revised. The experimental estimation of the spin Hall angle ($\theta_{SH} = 0.11$) was computed from the experimental efficiency obtained from harmonic Hall voltage measurements, $\frac{B_{SH}}{j} = \frac{\hbar\theta_{SH}}{2|e|M_s L_z} \approx 6.9 \times 10^{-7}$mT/Acm^{-2}, where j represents the current density in the Pt layer and L_z the thickness of the Co layer. Note that although this more precise estimation of current flowing in the Pt/Co bilayer would result in a different value of the spin Hall angle, our quantitative description of their experiments[5] presented in the main text would be equally accurate if this value of θ_{SH} is adopted as input for the modeling along with the corresponding current density in the Pt layer.

Again, the Oersted field ($\vec{B}_{Oe}$) considering the applied current and its spatial distribution (Fig. S.8) clearly indicates that its influence in the current-induced magnetization switching

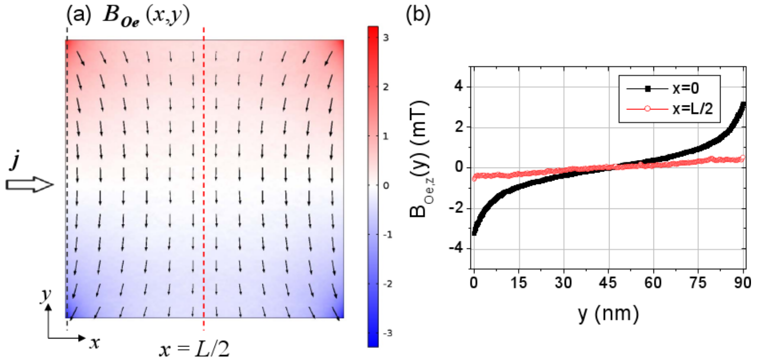

FIG. 9: (Color online) Oersted field $\vec{B}_{Oe}$ in the Co layer computed by COMSOL[24] corresponding to Fig. S.8. The injected current density through the Pt far from the Co layer is $j_a = 10^{12}$A/m^2.

is negligible (see Fig. S.9). $\vec{B}_{Oe}$ in the Co layer reaches its higher magnitude at top ($y = L$) and bottom ($y = 0$) edges where $|B_{Oe,max}| \approx 3$mT for $j_a = 10^{12}$A/m^2, which would result in $|B_{Oe,max}| \approx 12$mT for $j_a = 4 \times 10^{12}$A/m^2. As in the previous case, this value is ≈ 20 times smaller than in-plane and SHE effective fields, so it does not modify significantly the results, as it was confirmed by full micromagnetic simulations including it for some tested cases.

VII. DISCUSSION OF THE MICROMAGNETIC PARAMETERS

In this study we focus our attention in ultrathin FM Co layer (0.6nm) sandwiched between a HM Pt with thickness in the range $3-5$nm and capped by AlO. The material parameters considered as inputs for the modeling are the saturation magnetization M_s, the exchange stiffness constant A, the uniaxial PMA constant K_u, the Gilbert damping α, the spin Hall angle θ_{SH} and the DMI parameter D. Due to the atomic scale thickness of the FM Co layer, the polarization factor P of the spin current flowing through it is assumed to be negligible in agreement with several experiments[9,13–15], and therefore, both the conventional adiabatic and non-adiabatic STTs along with the FL-SOT due to the Rashba are assumed to be negligible, and these effects are not taken into account in the results of the main text. Nevertheless, several micromagnetic tests were performed including the adiabatic and non-adiabatic STTs with $P = 0.5$ and $\beta = 0.3$. Although these STTs introduce a tiny delay in the CIMS (see Fig. S3), they do not modify substantially the CIMS driven by the SHE.

The SL-SOT due to the Rashba has the same symmetry than the corresponding spin

Hall SL-SOT. However, here we are neglecting its effect based on direct experimental measurements[3] and also on the fact that it would need finite contribution from the conventional STTs with either $P < 0$ or a negative non-adiabatic parameter ($\beta < 0$) to explain the current-driven DW dynamics against the electron flow. Although some theoretical studies have suggested $P < 0$ or $\beta < 0$, no experimental evidence has been presented so far. Moreover, our assumption is also justified by the fact that this Rashba SL-SOT would be also proportional to the spin polarization of the current P which is assumed to be vanishingly small for the atomistic scale of the Co thickness[7,9,13–15]. Therefore, although we do not exclude a finite Rashba-SL-SOT contribution which could be explained by future theoretical developments, in the present work we only consider the spin Hall effect as the dominant SL-SOT contribution. Indeed, the SL-SOT due to the SHE along with the DMI explain by themselves both the current-induced magnetization switching (CIMS) and the current-driven DW motion (CIDWM), not only for the system of interest here (Pt/Co/AlO) but also for other systems such as Pt/CoFe/MgO and Ta/CoFe/MgO among others[7,9].

Although at present the experimentally deduced parameters (M_s, A, K_u, α for the Co and θ_{SH} for the Pt) for Pt/Co/AlO stacks are very similar (in the same order of magnitude and differing at most by a factor of $< 30\%$), they depict some scattered values depending on the authors and experiments. M_s can be measured by vibrating sample magnetometry (VSM). Miron et al.[1,6] have addressed $M_s = 1.1 \times 10^6$A/m, but the same group have measured $M_s = 8.7 \times 10^5$A/m for similar Pt(3)/Co(0.6)/AlO(2) systems but patterned with $L = 90$nm in-plane dimensions[5], with numbers in parenthesis representing the thickness in nm. For a unpatterned Pt(4)/Co(0.8)/AlO(1.9) films, Franken et al.[11] measured $M_s = 1.17 \times 10^5$A/m similar to the first measured value by Miron et al.[1,6], suggesting that also they obtained $M_s = 1.1 \times 10^6$A/m for unpatterned films. Liu et al.[3] reported $M_s = 1.0 \times 10^6$A/m for an Pt(3)/Co(0.6)/AlO patterned Hall bar with dimensions $200\mu\text{m} \times 20\mu\text{m}$. In this work, where we are considering patterned square dots with $L \sim 90$nm, we assume $M_s = 8.7 \times 10^5$A/m for Pt(3)/Co(0.6)/AlO(2) as estimated experimentally in ref.[5] for the same dimensions.

The exchange constant A for bulk Co ranges from 10^{-11}J/m to 3×10^{-11}J/m depending on the literature. A recent estimation of $A \approx 10^{-11}$J/m was recently done by Emori et al.[9] by spin Hall magnetometry for a 0.6nm-thick CoFe ultrathin strip sandwiched between Ta and MgO layers. This spin Hall magnetometry has not been performed yet for untrathin Co layers between Pt and AlO. Several authors[16,21] use an intermediate value of

1.6×10^{-11}J/m for Pt/Co/AlO systems, which is the same value we are considering here to reproduce the experiments by Garello[5]. Some trials were also done with $A = 10^{-11}$J/m with analogous qualitative results, but the better quantitative agreement with the experiments for Pt/Co/AlO was achieved with $A = 1.6 \times 10^{-11}$J/m.

The magnitude of the uniaxial PMA constant K_u is commonly deduced from the measurement of in-plane field ($B_{sat} = \mu_0 H_{sat}$) needed to saturate the magnetization in the plane of the film. From this H_{sat}, the effective uniaxial anisotropy K_{eff} is deduced. K_{eff} includes contributions from the uniaxial PMA K_u and from the out-of-plane contribution shape anisotropy, $K_{eff} = K_u - \frac{1}{2}N_z\mu_0 M_s^2$ with N_z the out-of-plane magnetostatic factor by Aharoni[20]. The typical values for K_u obtained from $K_{eff} = K_u - \frac{1}{2}N_z\mu_0 M_s^2$ range from 1.1×10^6J/m^3 (see[17] and[11] for unpatterned films) to 8.9×10^5J/m^3 as would be deduced from ref.[5] for a patterned square dot with $L \sim 90$nm. Although this way to estimate K_u could be justified as a first approach, a better estimation can be performed for more accurately obtaining K_u. Note that using $K_{eff} = K_u - \frac{1}{2}N_z\mu_0 M_s^2$ implies assuming $N_z \approx 1$ which could be justified if the sample depicted uniform-magnetization. However, this is not the case due to the finite DMI, and the full 3D space dependence of the demagnetizing field should be taken into account. Moreover, the value of $B_{sat} = \mu_0 H_{sat}$ could be influenced by the DMI, and it would play a significant role, specially for patterned dots at the nanoscale. We have confirmed these facts by full micromagnetic modeling: the saturating in-plane field B_{sat} for a square dot with $L = 90$nm differs when considering or not the effect of DMI. The finite DMI considered here ($D = 1.4$mJ/m^2, justified below from independent measurements) increases the value of B_{sat} needed to completely align the magnetization in-plane with respect to the $D = 0$ case by more than $\approx 20\%$. Using the experimentally measured value for the saturation magnetization $M_s = 8.7 \times 10^5$A/m and the DMI parameter $D = 1.4$mJ/m^2 (justified below), a saturating in-plane field of $B_{sat} \approx 1$T was micromagnetically obtained with $K_u = 8.0 \times 10^5$J/m^3, which is exactly the observable in the experiments[5]. Therefore, value of $K_u = 8.0 \times 10^5$J/m^3 was used to reproduce the experimental results by Garello et al.[5].

The Gilbert damping of bulk Co is $\alpha \sim 0.01$. However, this bulk value is not a proper value when an ultrathin Co layer is sandwiched between a HM and an oxide, and several works use values in the range from $\alpha = 0.1$ to $\alpha = 0.5$[16,17,21] in ultrathin Pt/Co/AlO stacks. Recent experiments using time-resolved magneto-optical Kerr effect by Schellekens et al.[19]

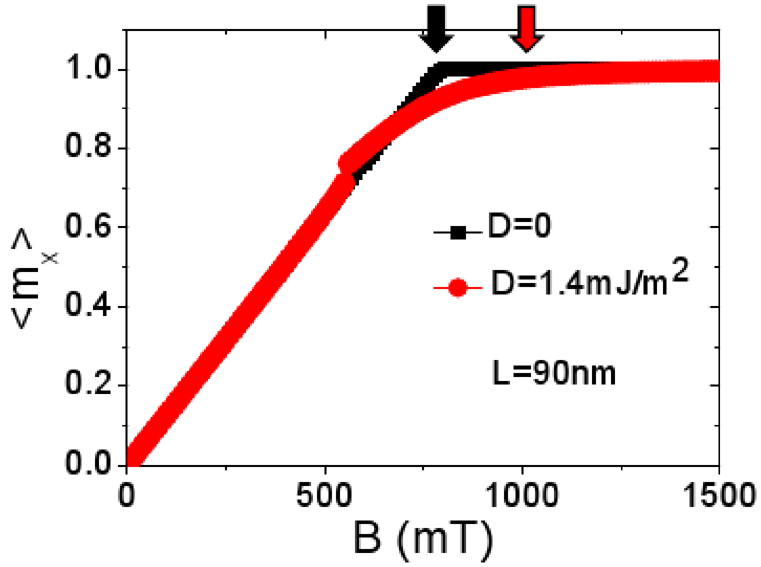

FIG. 10: (Color online) Full micromagnetic study of the DMI influence on the saturating in-plane field in square FM dot with $L = 90$nm and $L_z = 0.6$nm. The fixed material parameters are $A = 1.6 \times 10^{-11}$J/m, $M_s = 8.7 \times 10^5$A/m and $K_u = 8.0 \times 10^5$J/m^3, and equilibrium states for each applied field $\vec{B} = B\vec{u}_x$ are computed for both $D = 0$ and $D = 1.4$mJ/m^2. In the absence of the DMI ($D = 0$) a saturating field (defined as the field at which the in-plane magnetization along the in-plane field ($< m_x >$) reaches 0.99) is $B_{sat} \approx 0.76$T. However, in the presence of DMI ($D = 1.4$mJ/m^2) the saturating field is $B_{sat} \approx 1$T, exactly the same value as measured in the experiments of ref.[5].

for this Pt/Co/AlO stacks pointed out a dependence of α on the AlO, with values from 0.11 to 0.28. For our analysis a value of $\alpha = 0.3$ have been considered based in these experimental measurements and in others analyzing the field-driven DW motion[25].

The value of the spin Hall angle for ultrathin Pt/Co/AlO stacks have been ranged from $0.056 < \theta_{SH} < 0.16$[2,4]. An intermediate value of $\theta_{SH} = 0.11$ in this range has been selected for our study in main text, which is also the same as the one experimentally deduced by Garello et al.[5]. As it was already mentioned, this value was estimated from the SHE efficiency $\frac{H_{SH}}{j} = \frac{\hbar\theta_{SH}}{2e\mu_0 M_s L_z}$ by considering that the electric current flows uniformly through the Pt/Co bilayer. As mentioned above (Fig. S.4), if the different electrical resistivity of the Pt and Co layers were taken into account, only 64% of the current would be flowing through the Pt, and therefore, this would result in a different spin Hall angle. In order to reproduce the experimental data by Garello et al.[5] by considering uniform current through both the Pt

and Co layer, we have use their experimentally deduced value $\theta_{SH} = 0.11$. Note also that a very close value was also recently deduced for Pt $\theta_{SH} = 0.098$ by Ryu et al.[10].

Finally, it remains to justify the assumed value for the DMI parameter. At the moment, the measurements of this parameter are still very few. For instance, using spin Hall magnetometry, Emori et al.[9] obtained a value of $D = 1.2\text{mJ/m}^2$ from the efficiency of the current-induced DW motion along a Pt(3)/CoFe(0.6)/MgO. More recently, Stashkevich et al.[26] have estimated experimentally the same value ($D = 1.2\text{mJ/m}^2$) by means of Brillouin spectroscopy in a Py/Pt bilayer, whereas a value of $D = 1.38\text{mJ/m}^2$ was experimentally obtained for Pt/Co/AlO by Brillouin light scattering[18]. Based on this measurement, a value of $D = 1.4\text{mJ/m}^2$ have been used in our qualitative and quantitative analysis in the main text. In short, all our inputs for the material parameters are well justified within the uncertainty and the scattered of the experimentally deduced values.

VIII. SINGLE DOMAIN MODEL AND MICROMAGNETIC ANALYSIS OF THE CURRENT-INDUCED MAGNETIZATION SWITCHING AS FUNCTION OF THE DMI PARAMETER AND THE SPIN HALL ANGLE

As discussed in former Sec. VII, the spin Hall angle θ_{SH} and the DMI parameter D for Pt systems can vary depending on the literature. Here, we analyze the CIMS for different values of θ_{SH} and D within the range of available data: $0.05 \leq \theta_{SH} \leq 0.16$ and $0.8\text{mJ/m}^2 \leq D \leq 2.0\text{mJ/m}^2$. We focus in the study of Fig. 6 of the main text, which was performed to reproduce the experimental results by Garello et al.[5] (see Fig. 2d therein[5]). A square of $L = 90$nm and $L_z = 0.6$nm with fixed $M_s = 8.7 \times 10^5$A/m, $A = 1.6 \times 10^{-11}$J/m, $K_u = 8.7 \times 10^5\text{J/m}^3$ and $\alpha = 0.3$ is considered, and the CIMS from $\uparrow$ to $\downarrow$ is studied at zero temperature under positive fields $B > 0$ and positive current pulses with $t_R = t_F = 200$ps and $\tau = 20$ps and different amplitudes. The SDM results under four different values of θ_{SH} are shown in Fig. S. 11. As expected, the critical current to achieve switching decreases as the longitudinal field increases, and the transition from no-switching to switching is reduced as the spin Hall angle increases. A comparison to the experiments results of Fig. 2d in[5] indicates that the SDM is not able to reproduce experimental observations in the range of experimentally deduced values for θ_{SH}. Note that even with a unrealistic high $\theta_{SH} = 0.2$, the SDM is neither able to reproduce the experimental results, as it was shown in Fig. 1g

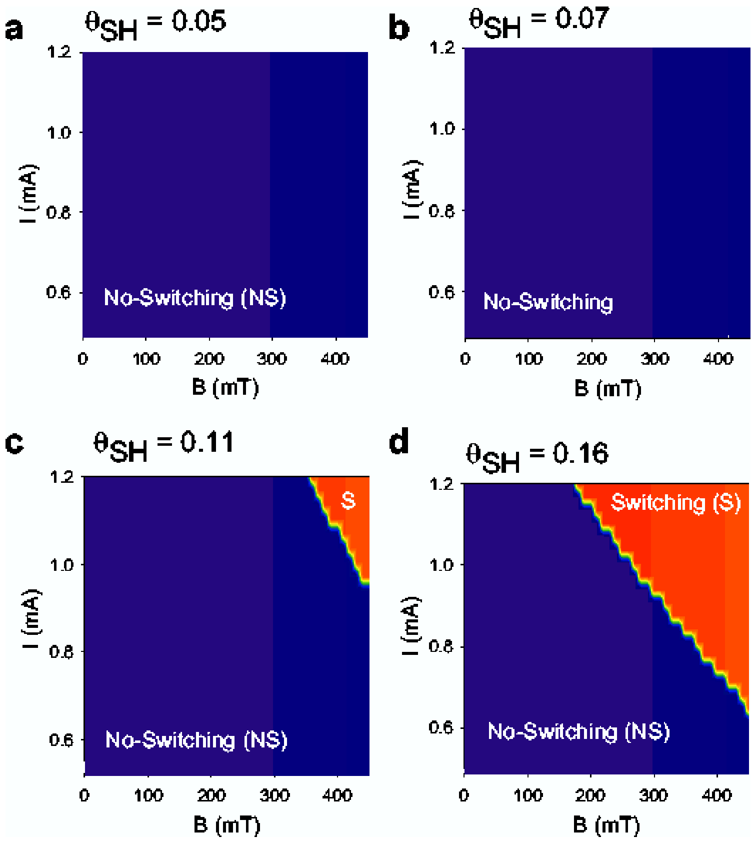

FIG. 11: (Color online) Current-induced magnetization switching as function of the spin Hall angle θ_{SH} computed in the framework of the Single Domain Model (SDM). Each diagram shows the Switching (S) / No-Switching (NS) phase diagram starting from the up state ($\uparrow$) as function of I and B. All inputs are given in the text.

in the main text.

The same analysis as in Fig. S.11 was also performed with full micromagnetic simulations (μM), where the spacial dependence of the magnetization and effective fields along with the conventional exchange and the Dzyaloshinskii-Moriya interactions can be naturally evaluated. The μM results for θ_{SH} : $0.05, 0.11, 0.16$ and D : $0.8\text{mJ/m}^2, 1.4\text{mJ/m}^2, 2.0\text{mJ/m}^2$ are depicted in the Fig. S.12. For the smallest spin Hall angle ($\theta_{SH} = 0.05$), the agreement with the experimental results is not achieved even with the highest DMI parameter ($D = 2.0\text{mJ/m}^2$). For $\theta_{SH} = 0.16$, a good agreement is observed when $D = 0.8\text{mJ/m}^2$. For the intermediate value ($D = 1.4\text{mJ/m}^2$), a good agreement is achieved with the intermediate value of $\theta_{SH} = 0.11$, the same as inferred in[5].

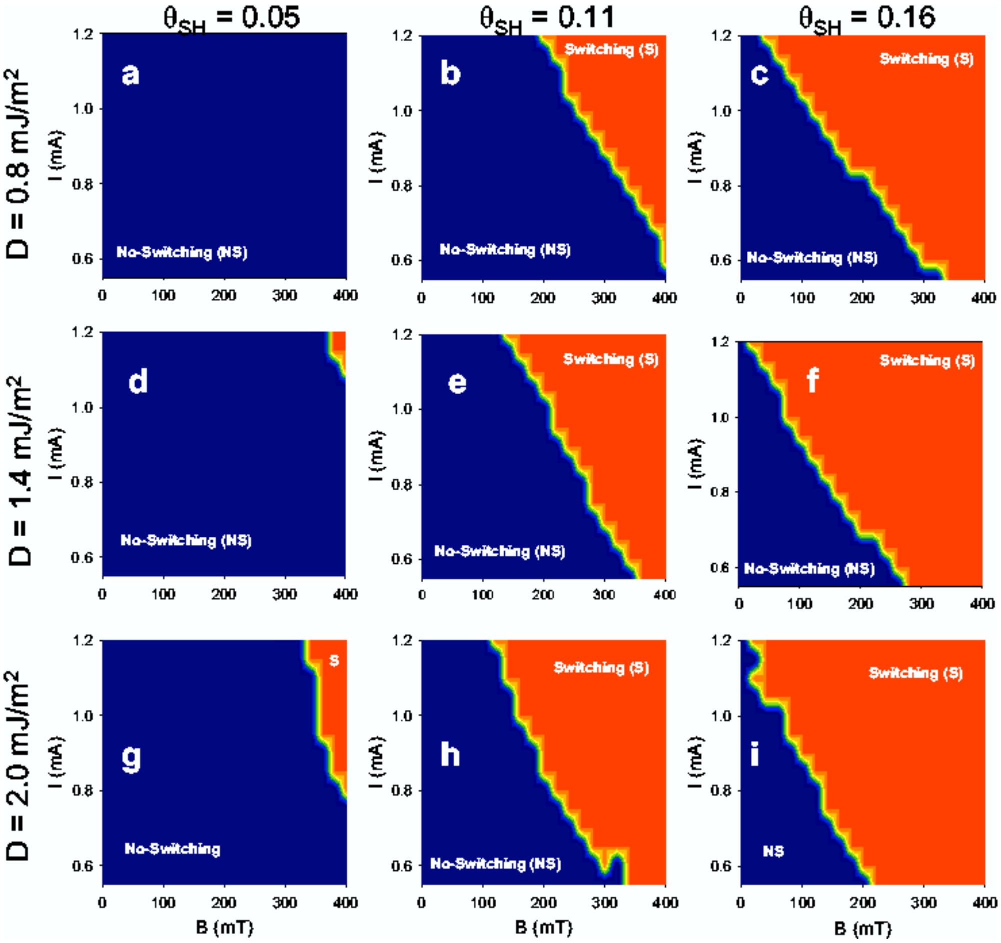

FIG. 12: (Color online) Current-induced magnetization switching as function of the spin Hall angle θ_{SH} computed in the framework of the micromagnetic model (μM). Each graph shows the Switching / No-Switching phase diagram starting from the up state (↑) as function of I and B corresponding to the experimental ranges experimentally studied in Fig. 2d of[5]. All inputs are given in the text.

IX. ANALYSIS OF POSSIBLE SOURCES OF DISCREPANCY WITH EXPERIMENTAL DATA

Although our μM simulations describe the experimental data by Garello et al.[5] with a quantitative accuracy that is well within any reasonable expectation in experimental physics, the exact reproduction of their experiments is beyond the scope of the present work. Here, possible sources of these minor discrepancies between their experimental and our μM results can be enumerated as follows:

i) As it is usually considered in experiments[5,7], in our μM study the current is assumed to be uniformly distributed throw the Pt/Co bilayer. However, the tabulated resistivity of Co is $\rho(Co) = 62.4 \times 10^{-9}\Omega$m whereas $\rho(Pt) = 105 \times 10^{-9}\Omega$m for Pt at room temperature. In the studied stack the current density is not uniformly flowing in the Pt/Co bilayer, and a simple estimation confirmed by COMSOL[24] simulations indicates that even for the small Co thickness (0.6nm) as compared to the Pt layer (3nm), indeed the 36% of the current would flow through the Co layer. This non-uniform distribution of the current would reduce the electrical current in the Pt and the SHE efficiency, and it would result in a smaller spin Hall angle. If the different electrical resistivity of the Pt and Co layers were taken into account, only 64% of the current would be flowing through the Pt, and therefore, this would result in a smaller spin Hall angle $\theta_{SH} = 0.07$, which is precisely the value deduced by Liu et al.[3].

ii) On the other hand, it is also known that the resistivity also depends on the magnetic texture[27], and therefore, it is expected that the resistivity of the Co layer increases as soon as the DW is nucleated with respect to the quasi-uniform state. Contrary to the pure electrical resistivity, this magneto-resistive effect would increase the current that flows through the Pt in detriment to the Co layer, resulting in a net increase of the SHE.

iii) The non-uniform current along the x-axis would also generate a non-uniform Oersted field $\vec{B}_{Oe}$ in the Pt/Co bilayer. This Oersted field reaches its maximum value at the transversal edges of the Co sample ($y = 0$ and $y = L$) with strong out-of-plane components of different polarities at these bottom and top edges depending on the current direction. For instance, starting from the state mainly magnetized along the positive z-axis and injecting a positive current ($j > 0$) under the presence of a positive field ($B > 0$), the Oersted field opposes to the out-of-plane component of effective SHE at the top-left corner ($H_{SH,z}(0, L)$), whereas it supports the out-of-plane component of the effective SHE field in the bottom-left corner ($H_{SH,z}(0, 0)$). Assuming that the electric current flows uniformly through the Pt/Co section, the maximum value for the Oersted field is $\sim$ 11mT pointing out-of-plane at the top and and bottom edges, estimated for $j_a = 3.7 \times 10^{12}$A/m^2. A similar maximum value was computed by a COMSOL simulation[24] considering the different resistivities of the Co and Pt (see Fig. S.5). These values are a factor of 20 smaller than the z component SHE effective field ($H_{SH,z}$) in the top and bottom corners at the left edge of the sample, and therefore, the Oersted field does not have enough magnitude to modify the switching reversal described in the main text. Indeed, indistinguishable micromagnetic results from the ones depicted in

Fig. 3 of the main text were obtained when considering the Oersted field.

iv) It has to be taken into account that in the Co layer the thickness is only three atomic layers, and due to the fabrication process there must be a significant random disorder at the Pt/Co interface. This disorder must result in a random dispersion of some of the material parameters, such as K_u, D and θ_{SH}.

v) Finally, Joule heating effects due to the electric current flowing through the Pt and/or the Co layers may also play a role on the current induced magnetization switching, for instance by modifying the nominal values of M_s, A and K_u.

Although all these effects (non-uniform distribution of the current due to both the electric and magnetic effects, the Oersted field, the random dispersion of the material parameters due to the disorder at the Pt/Co and Co/AlO interfaces and Joule heating) should be taken into account for extremely precise estimations of the spin Hall angle and the DMI parameter, they are second-order contributions which would not modify the qualitative reversal mechanism addressed here. Refining the model to include them is however, beyond the scope of this work. Indeed, analogous reversal mechanisms consisting on DW nucleation and subsequent DW propagation were also observed under the presence of the edge roughness and thermal effects, which clearly supports the universality of the switching mechanism for strong DMI systems.

[1] I. M. Miron, K. Garello, G. Gaudin, P.J. Zermatten, M. V. Costache, S. Auffret, S. Bandiera, B. Rodmacq and P. Gambardella. Nature. 476, 189 (2011).

[2] L. Liu, T. Moriyama, D. C. Ralph and R. A. Buhrman. Phys Rev. Lett. 106, 036601 (2011).

[3] L. Liu, O. J. Lee, T. J. Gudmundsen, D. C. Ralph and R. A. Buhrman. Phys Rev. Lett. 109, 096602 (2012).

[4] K. Garello, I. M. Miron, C. O. Avci, F. Freimuth, Y. Mokrousov, S. Bluugel, S. Auffret, O. Boulle, G. Gaudin, and P. Gambardella, Nature Nanotechnol. 8, 587 (2013).

[5] K. Garello, C. O. Avci, I. M. Miron, M. Baumgartner, A. Ghosh, S. Auffret, O. Boulle, G. Gaudin, and P. Gambardella. Appl. Phys. Lett. 105, 212402 (2014).

[6] I. M. Miron, T. Moore, H. Szambolics, L. D. Buda-Prejbeanu, S. Auffret, B. Rodmacq, S. Pizzini, J. Vogel, M. Bonfim, A. Schuhl and G. Gaudin. Nat. Mat. 10, 419 (2011).

[7] S. Emori, U. Bauer, S.-M. Ahn, E. Martinez, and G. S. D. Beach. Nature Mat. 12, 611 (2013).

[8] K.-S. Ryu, L. Thomas, S.-H. Yang, and S. Parkin, Nat. Nanotechnol. 8, 527 (2013).

[9] S. Emori, E. Martinez, U. Bauer, S.-M. Ahn, and G. S. D. Beach. arXiv:1308.1432 (2013).

[10] K.-S. Ryu, S.-H. Yang, L. Thomas, and S. Parkin, Nat. Comm. 5, 3910 (2014).

[11] J. H. Franken, M. Herps, H. J. M. Swagten and B. Koopmans, Scientific Reports. 4 , 5248, DOI: 10.1038 (2014).

[12] A. Thiaville, Y. Nakatani, J. Miltat, and Y. Suzuki. Europhys. Lett., 69 (6) 990996 (2005).

[13] M. Cormier, A. Mougin, J. Ferre, A. Thiaville, N. Charpentier, F. Piechon, R. Weil, V. Baltz, and B. Rodmacq. Phys. Rev. B 81, 024407 (2010).

[14] S. Emori and G.S.D. Beach J. Phys.: Condens. Matter 24 024214 (2012).

[15] H. Tanigawa, T. Suzuki, S. Fukami, K. Suemitsu, N. Ohshima and E. Kariyada. Appl. Phys. Lett. 102, 152410 (2013).

[16] J. Sampaio, V. Cros, S. Thiaville and A. Fert. Nat. Nanotech. 8, 839 (2013).

[17] S. Pizzini, J. Vogel, S. Rohart, L. D. Buda-Prejbeanu, E. Ju, O. Boulle, I. M. Miron, C. K. Safeer, S. Auffret, G. Gaudin, and A. Thiaville. Phys. Rev. Lett. 113, 047203 (2014).

[18] A value of the DMI parameter $D = 1.38\text{mJ/m}^2$ was recently experimentally obtained for Pt/Co/AlO by Brillouin light scattering. see J. Cho, J. Kim, N. Kim, S. Lee, A. Solignac, Y. Yin, H. Swagten, B. Koopmans. Abstract CB-12 of HMM 2014: Determination of the interfacial Dzyaloshinskii-Moriya interaction energy density by Brillouin light scattering.

[19] A. J. Schellekens, L. Deen, D. Wang, J. T. Kohlhepp, H. J. M. Swagten, and B. Koopmans. App. Phys. Lett. 102, 082405 (2013).

[20] A. Aharoni. J. Appl. Phys. 83, 3432 (1998).

[21] O. Boulle, S. Rohart, L. D. Buda-Prejbeanu, E. Ju, I. M. Miron, S. Pizzini, J. Vogel, G. Gaudin, and A. Thiaville. Phys. Rev. Lett. 111, 217203 (2013)

[22] E. Martinez, S. Emori, N. Perez, L. Torres and G. S. D. Beach. J. Appl. Phys. 115, 213909 (2014).

[23] E. Martinez and O. Alejos. J. Appl. Phys. 116, 023909 (2014).

[24] COMSOL Multiphysics. www.comsol.com.

[25] P. J. Metaxas Phys. Rev. Lett. 99, 217208 (2007).

[26] A. A. Stashkevich, M. Belmeguenai, Y. Roussigne, S. M. Cherif, M. Kostylev, M. Gaborm D. Lacour, C. Tiusan and M. Hehn. Non-reciprocity of spin wave propagation induced by the

interface Dzyaloshinskii-Moriya interaction in Py/Pt film structures. arXiv:1411.1684v1, (2014).

[27] J. H. Franken, M. Hoeijmakers, H. J. M. Swagten and B. Koopmans, Phys. Rev. Lett. 108, 037205 (2012).
